# Inhibition of Nox4‐dependent ROS signaling attenuates prostate fibroblast activation and abrogates stromal‐mediated protumorigenic interactions

**DOI:** 10.1002/ijc.31316

**Published:** 2018-03-01

**Authors:** Natalie Sampson, Elena Brunner, Anja Weber, Martin Puhr, Georg Schäfer, Cedric Szyndralewiez, Helmut Klocker

**Affiliations:** ^1^ Department of Urology, Division of Experimental Urology Medical University of Innsbruck Innsbruck Austria; ^2^ Division of Pathology Medical University of Innsbruck Innsbruck Austria; ^3^ Genkyotex S.A. Geneva Switzerland

**Keywords:** prostate cancer, cancer‐associated fibroblast, microenvironment, reactive oxygen species, transforming growth factor beta, *in situ* hybridization

## Abstract

Carcinoma‐associated fibroblasts (CAFs) play a key onco‐supportive role during prostate cancer (PCa) development and progression. We previously reported that the reactive oxygen species (ROS)‐producing enzyme NADPH oxidase 4 (Nox4) is essential for TGFβ1‐mediated activation of primary prostate human fibroblasts to a CAF‐like phenotype. This study aimed to further investigate the functional relevance of prostatic Nox4 and determine whether pharmacological inhibition of stromal Nox4 abrogates paracrine‐mediated PCa‐relevant processes. RNA *in situ* hybridization revealed significantly elevated Nox4 mRNA levels predominantly in the peri‐tumoral stroma of clinical PCa with intense stromal Nox4 staining adjacent to tumor foci expressing abundant TGFβ protein levels. At pharmacologically relevant concentrations, the Nox1/Nox4 inhibitor GKT137831 attenuated ROS production, CAF‐associated marker expression and migration of TGFβ1‐activated but not nonactivated primary human prostate fibroblasts. Similar effects were obtained upon shRNA‐mediated silencing of Nox4 but not Nox1 indicating that GKT137831 primarily abrogates TGFβ1‐driven fibroblast activation via Nox4 inhibition. Moreover, inhibiting stromal Nox4 abrogated the enhanced proliferation and migration of PCa cell lines induced by TGFβ1‐activated prostate fibroblast conditioned media. These effects were not restricted to recombinant TGFβ1 as conditioned media from PCa cell lines endogenously secreting high TGFβ1 levels induced fibroblast activation in a stromal Nox4‐ and TGFβ receptor‐dependent manner. Importantly, GKT137831 also attenuated PCa cell‐driven fibroblast activation. Collectively, these findings suggest the TGFβ‐Nox4 signaling axis is a key interface to dysregulated reciprocal stromal–epithelial interactions in PCa pathophysiology and provide a strong rationale for further investigating the applicability of Nox4 inhibition as a stromal‐targeted approach to complement current PCa treatment modalities.

## Abstract 2

**What's new?**

While the tumor microenvironment plays a critical role in prostate adenocarcinoma pathogenesis, underlying mechanisms remain unclear. This study demonstrates the functional relevance of elevated expression of Nox4—which is a major source of cellular reactive oxygen species (ROS)—in the tumor microenvironment and downstream consequences on prostate cancer (PCa)‐relevant processes. Findings identify stromal Nox4 as a central mediator of reciprocal epithelial‐stromal cell crosstalk, fibroblast activation and stromal‐driven tumor (cell)‐promoting hallmarks. The data thus provide a strong mechanistic rationale for therapeutic inhibition of Nox4 as a stromal‐targeted approach to complement current treatment strategies for PCa and restore dysregulated reciprocal stromal–epithelial interactions.

**Abbreviations**
AR: androgen receptor
bFGF: basic fibroblast growth factor
CAF: carcinoma associated prostate fibroblast
CAT: catalase
CM: conditioned media
CNN1: calponin
COMP: cartilage oligomeric matrix protein
ctFCS: charcoal treated fetal calf serum
FAP: fibroblast activation protein
GAPDH: glyceraldehyde 3‐phosphate dehydrogenase
GEO: gene expression omnibus
HMBS: hydroxymethylbilane synthase
IGFBP3: insulin‐like growth factor binding protein 3
Nox: NADPH oxidase
PCa: prostate cancer
qPCR: quantitative real time PCR
ROS: reactive oxygen species
SERPINE1: serpin family E member 1
shRNA: short hairpin RNA
SMA: alpha smooth muscle actin
TBP: TATA binding protein
TGFβ1: transforming growth factor beta

Prostate cancer (PCa) is the second leading cause of male cancer‐related death in Western societies.^1^ While epithelial in origin, the tumor microenvironment plays a critical role in prostate adenocarcinoma pathogenesis, for example, stromal signaling is required for tumor initiation, tumor cell proliferation, angiogenesis, metastasis and diminishes therapy response.^2^, ^3^, ^4^ These protumorigenic actions of the tumor microenvironment are largely mediated via the secretion of paracrine‐acting factors, including chemokines, cytokines, growth factors, extracellular matrix (ECM) components and ECM remodeling enzymes.^5^ The importance of the tumor‐associated stroma as a driver of PCa progression and independent predictor of PCa prognosis is underscored by clinical data.^6^, ^7^ Thus, there is considerable interest in targeting the tumor microenvironment as a therapeutic strategy for PCa.

The tumor‐adjacent stroma is particularly enriched with activated fibroblasts (termed cancer‐associated fibroblasts, CAFs) as defined by their expression of fibroblast activation protein (FAP) and alpha smooth muscle actin (SMA).^8^ CAFs are similar to those during inflammation and wound healing and represent a heterogeneous stromal cell population with distinct yet poorly defined subtypes exhibiting well‐documented protumorigenic but also tumor‐inhibitory properties.^9^ These differences most likely reflect distinct CAF cellular origins and activating stimuli.^9^ While CAFs may originate from multiple sources (e.g., pericytes, endothelial cells and bone marrow‐derived circulating precursors), growing evidence indicates that the tumor‐associated stroma predominantly derives from precursors in the local tumor microenvironment.^10^ In particular, local resident fibroblasts are thought to be activated via tumor cell‐derived soluble factors of which transforming growth factor beta 1 (TGFβ1) is the most characterized and activates prostatic fibroblasts to a CAF‐like phenotype *in vitro* and *in vivo*.^2^, ^8^ Notably, circulating TGFβ1 levels positively correlate with PCa risk, rapid disease progression and poor outcome.^11^, ^12^

At excessive levels, reactive oxygen species (ROS) can damage DNA and other cellular components. However, ROS are also key regulators of diverse physiological processes (e.g., proliferation, apoptosis and differentiation) via reversible thiol modification of redox‐sensitive proteins, resulting in conformational changes that alter enzymatic activity (kinases and phosphatases) or DNA binding activity of transcription factors (e.g., NFκB and AP‐1).^13^ NADPH oxidase (Nox) enzymes are a major source of cellular ROS and thus key regulators of inter‐ and intracellular redox signaling.^14^ The Nox family comprises seven members (Nox1–5 and Duox1–2) that catalyze the transfer of electrons from molecular oxygen across biological membranes using NADPH as electron donor thereby generating superoxide. Nox4 is unique amongst Nox enzymes as it is constitutively active with hydrogen peroxide (H_2_O_2_) being the primary ROS detectable.^15^, ^16^ ROS play a central role in fibroblast activation.^17^ In particular, we previously demonstrated a pivotal function of Nox4‐derived ROS during TGFβ1‐induced activation of prostate fibroblasts most likely via redox‐dependent phosphorylation of c‐Jun N‐terminal kinase (JNK).^18^ More recently, we and others showed that Nox4 expression is higher in PCa patients that experience biochemical relapse following radical prostatectomy and in patients with decreased PCa‐specific survival.^19^, ^20^ Thus, this study aimed to further investigate the functional relevance of prostatic Nox4 and assess whether pharmacological inhibition of stromal Nox4 may represent a potential PCa therapeutic strategy.

## Materials and Methods

### Reagents

Reagents were from Sigma‐Aldrich (Vienna, Austria) unless otherwise specified. Human recombinant TGFβ1 was from R&D Systems (Minneapolis, MN). Inhibitors and concentrations employed were: TGFβ type 1 receptor inhibitor SB431542 (1 μM, Tocris Bioscience, Bristol, UK), dual Nox1/Nox4 inhibitor GKT137831 (30 μM) was a gift from GenKyoTex S.A. (Geneva, Switzerland). Antibodies employed were pan TGFβ and IGFBP3 (R&D Systems), SMA and CNN1 (Sigma), GAPDH (Merck Millipore, Vienna, Austria), Nox4 (Abcam, Cambridge, UK) and FAP (Biorbyt, Cambridge, UK).

### Cell culture and primary human prostate fibroblast isolation

Prostate epithelial cell lines were obtained from American Type Culture Collection (ATCC; Rockville, MD) and maintained according to the distributor's instructions. All cells were maintained in a humidified atmosphere at 37°C with 5% CO_2_. Mycoplasma‐free human primary prostatic fibroblasts were established via an outgrowth method from prostate organoids as described previously^21^ from histologically verified benign prostate regions from men undergoing radical prostatectomy for PCa. The use of patient material for cell isolation was approved by the ethics committee of the Medical University of Innsbruck (UN4668 311/4.12) and all patients gave prior written informed consent. Fibroblasts were maintained in DMEM (Lonza, Cologne, Germany) containing 10% fetal calf serum (FCS, PAN Biotech, Aidenbach, Germany), 1% penicillin/streptomycin and 2% GlutaMAX™ (GIBCO, Fisher Scientific, Vienna, Austria) and used at passage 8 or lower. For TGFβ1 activation, fibroblasts were seeded in phenol red‐free DMEM (GIBCO) containing 2.5% charcoal treated FCS (ctFCS; PAN Biotech) and allowed to adhere overnight. Cells were subsequently treated with 1 ng/ml basic fibroblast growth factor (bFGF) as mock control to maintain the fibroblast phenotype or 1 ng/ml TGFβ1 in phenol red‐free DMEM containing 1% ctFCS for the indicated duration. Where inhibitors were employed, cells were pretreated for 1 hr with the appropriate inhibitor or vehicle equivalent before treatment with bFGF or TGFβ1 as indicated. All experiments were performed at least three times with primary cells from different donors. In total, experiments herein employed fibroblasts from 33 different donors.

### TGFβ ELISA

TGFβ1 and TGFβ2 concentrations were determined in duplicate from 3 independently generated batches of prostate epithelial cell conditioned media (CM) using a TGFβ1 or TGFβ2 Quantikine ELISA kit according to the manufacturer's instructions (R&D Systems).

### Conditioned media generation and application

Fibroblasts were treated as indicated for 48 hr in DMEM supplemented with 1% ctFCS before rinsing in serum‐free DMEM and incubated for further 48 hr in serum‐free DMEM/0.1% BSA supplemented with 30 μM GKT137831 or DMSO equivalent. The supernatant was collected as conditioned media (CM). Non‐CM control media were generated from the same mastermix of serum‐free DMEM/0.1% BSA supplemented with 30 μM GKT137831 or DMSO equivalent but incubated for the same duration without cells. Prostate epithelial cells were seeded in 10 cm dishes in routine culture media diluted 1:1 with serum‐free DMEM/0.1% BSA. Once 80% confluent monolayers had formed, media was aspirated, cells washed in serum‐free DMEM and incubated for a further 72 hr in serum‐free DMEM/0.1% BSA. The supernatant was collected as CM. CM were centrifuged at 1000 rpm for 5 min and frozen in aliquots for later use. CM were normalized with fresh serum‐free DMEM/0.1% BSA according to the protein content of lysates from the corresponding cell monolayers as determined via BCA protein assay (Pierce, Thermo Scientific, Vienna, Austria). Normalized CM were diluted 2:1 with fresh serum‐free DMEM/0.1% BSA and applied to the indicated cells for the duration stated.

### Lentiviral‐mediated shRNA‐mediated silencing

Lentiviral particles were generated and fibroblasts infected as described.^18^ Nox1 and Nox4 shRNAs (GE Healthcare Dharmacon Vienna, Austria) employed were TRCN0000046085 and TRCN0000046086 (Nox1) and TRCN0000046090 and TRCN0000046089 (Nox4). Scramble shRNA vector (plasmid 1864 from Addgene, Cambridge, MA) was used as control.

### Proliferation assay

Cells seeded in triplicate in black 96‐well plates in phenol red‐free media were treated as indicated. Media were aspirated and cells lysed by freezing at −80°C. Upon thawing, 200 μl CyQuant lysis reagent (Invitrogen, Vienna, Austria) containing SybrGreen (final dilution 1/1000) was added. Following incubation for 30 min at 37°C, fluorescence was measured on a Tecan Genius Pro (Männedorf, Switzerland) and background intensity from cell‐free wells subtracted.

### ROS detection

Extracellular H_2_O_2_ was measured via Amplex Red assay (Molecular Probes, Fisher Scientific, Vienna, Austria) according to the manufacturer's instructions and fluorescence detected on a Tecan Genius Pro at 530 nm (excitation) and 590 nm (emission). The fluorescent intensity derived from replicate cells incubated with 250 U/ml catalase for 1 hr prior to incubation with the redox‐sensitive probe was subtracted from the appropriate experimental value and normalized against cell number as determined via SybrGreen staining (as above) of replica wells. Intracellular ROS production was measured using CM‐H_2_DCFDA (Molecular Probes) as described.^18^

### RNA isolation, cDNA synthesis and quantitative real‐time PCR (qPCR)

RNA isolation, cDNA synthesis and qPCR using Taqman^®^ gene expression assays were performed as described.^18^, ^22^ TaqMan^®^ gene expression assays (Applied Biosystems, Vienna, Austria) are listed in Supporting Information, Table 1. Data are presented as fold change in gene expression using the mathematical model ratio 2^−ΔΔCT^ 23 or as mean 2^−ΔCT^ defined as 2^−[Ct housekeeping gene − Ct target gene]^ thus providing an indication of the level of target gene expression relative to the moderately expressed housekeeping gene TATA binding protein (TBP).

### SDS PAGE and Western blotting

Preparation of cell lysates, normalization via BCA protein assay (Pierce, Thermo Scientific), SDS PAGE and Western blotting were performed as described.^18^ Membrane and cytosolic extracts from HEK293T cells stably overexpressing Nox4 or empty vector control were prepared as described.^24^

### Migration assays

Fibroblasts pretreated in 6‐well plates as indicated for 72 hr were trypsinized and resuspended in serum‐free DMEM (2.5 ml per well of 6 well plate). 0.5 ml was then transferred to the upper chamber of a 24‐well BD Fluoroblok transwell plate with an 8 μm pore size (BD Bioscience, Vienna, Austria). The lower chamber was filled with DMEM supplemented with 30% FCS. Cells were seeded in triplicate with an additional well without insert for cell number normalization. After 48 hr, migrated cells were stained using 2 μM Calcein AM in Hank's Balanced Salt Solution (HBSS, Lonza) for 1 hr at 37°C. After washing in HBSS, fluorescent images were acquired on a JuLI Smart Fluorescence Cell Imager (NanoEntek, CA), fluorescence measured on a Tecan Genius Pro plate reader in bottom read mode and background values from cell‐free wells subtracted. For analysis of stromal‐induced chemotaxis on prostate epithelial cells, subconfluent PCa cells were serum‐deprived for 24 hr before seeding into Fluoroblok transwell inserts as above at a density of 5 × 10^4^ (DU145) or 1 × 10^5^ (CWR22Rv1) per insert in 0.5 ml serum‐free DMEM/0.1% BSA. The lower chamber was filled with 0.7 ml normalized CM from fibroblasts treated as indicated and fresh serum‐free DMEM/0.1% BSA at a ratio of 2:1. PCa cells were seeded in triplicate with 1 additional well per CM without inserts for cell number normalization and migrated cells stained as above 24 hr (DU145) or 48 hr (CWR22Rv1) later.

### *In situ* hybridization and dual immunohistochemistry (IHC)

Formalin‐fixed paraffin‐embedded (FFPE) primary tumor specimens were obtained from previously untreated patients who had undergone radical prostatectomy at the Department of Urology, Innsbruck Medical University after PCa diagnosis in a PSA early cancer detection program.^25^ Use of patients’ samples was approved by the ethics committee of the Innsbruck Medical University (Study no. AM 3174 including amendment 2) and all patients gave written informed consent. The tissue microarray (TMA) employed herein has been described in detail previously.^26^ Nox1 and Nox4 *in situ* hybridization (ISH) were performed using the RNAscope 2.5 HD Red kit according to the manufacturer's instruction (Advanced Cell Diagnostics, Inc. Newark, CA). Positive (PPIB) and negative (DapB) control probes were hybridized in parallel for all experiments. For dual ISH‐IHC, FFPE sections were first subjected to ISH as above but with reduction of the protease treatment step to 20 min and increased duration of the Amp5 step to 45 min. After FastRed substrate detection, sections were rinsed in TBS, incubated 3 times for 10 min in 3% H_2_O_2_ before blocking (10 mg/ml BSA, 10% goat serum in TBS) for 1 hr and incubation with a pan TGFβ antibody (1:100, R&D Systems) overnight at 4°C. Control slides were incubated with an equivalent concentration of mouse IgG (Dako, Agilent Technologies, Santa Clara, CA). After washing, biotin‐labeled secondary antibodies (Dako) were added for 1 hr at RT, sections washed and extravadin–peroxidase complex added. After 30 min at RT, slides were washed and HIGHDEF yellow chromogen substrate added according to the manufacturer's instructions (Enzo Life Sciences AG, Lause, Switzerland). After rinsing, sections were counterstained in hematoxylin, dried and mounted in EcoMount (Biocare Medical, Concord, CA).

### Microscopy and imaging

Images were taken with a Zeiss Imager Z2 microscope (Zeiss, Vienna) equipped with a Pixelink PL‐B622‐CU camera (Canimpex Enterprises Ltd., Halifax, Canada). ISH staining of TMAs was evaluated by an uropathologist (G.S.) enabling separate quantification of epithelial and stromal Nox4 staining using an established semi‐quantitative “quick score” system combining the proportion of positive cells and the average staining intensity based on the established method first described by Detre (1995) *et al*.^27^ Briefly, quick score categories were based on both the proportion (denoted category A) and intensity (denoted category B) of positively stained cells. The proportion of positive cells (category A) was stratified into 4 groups (0 = negative, 1 = <30%, 2 = 30–60%, 3 = >60%). Average staining intensity (category B) corresponding to the present of negative, weak, intermediate and strong staining was given a score from 0 to 3, respectively. An average multiplicative quick score (category A × category B) was subsequently obtained from the 3 different benign or malignant tissue cores for each case. Total Nox1 and Nox4 expression were also quantified from images of prostate tissue sections taken at 40× magnification using ImageJ (v1.46r) via the YUV indexing option (*Y* = 0–255, *U* = 129–255, *V* = 136–255) and Huang threshold method, which yielded the best separation of ISH red signal from blue hematoxylin counterstain. Pixels above these threshold settings were analyzed using pixel size = 0–∞, circularity = 0–1.0 to include all objects. Ten fields of view each from tumor and adjacent benign areas were quantified for a total of 3 different patient specimens stained via ISH in independent experiments.

### Statistical analysis

Numerical data are presented as mean + SEM from at least three independent experiments using independent donors. Statistical evaluation was performed using a one‐way ANOVA followed by Tukey's post‐hoc test (ns, not significant; *p < 0.05; **p < 0.01, ***p < 0.001) using GraphPad Prism version 7 (La Jolla, CA). For nonparametric distributed data, Mann–Whitney *U* was used.

## Results

### Nox4 expression is elevated in the PCa‐associated stroma

We previously demonstrated that elevated Nox4 mRNA in clinical PCa positively correlates with biochemical relapse after radical prostatectomy.^19^ This study utilized macro‐dissected prostate tissue and thus did not permit identification of the cellular source of elevated Nox4 in PCa. Reliable detection of Nox proteins via immunohistochemistry (IHC) is problematic in many tissues,^28^ including the prostate (not shown). As Nox4 is constitutively active and primarily regulated at the mRNA level,^15^, ^16^ we utilized *in situ* hybridization (ISH) on prostate tissue sections and a PCa tissue microarray (TMA) to identify the cellular origin and semi‐quantify prostatic Nox4 mRNA levels using a highly specific assay with single mRNA transcript sensitivity (Supporting Information, Fig. S1).^29^ Nox4 mRNA was detected at low levels in both epithelial and stromal cells of the benign prostate (0.07% of cells in benign areas showed 1–2 mRNA transcript signals). Consistent with our previous findings that Nox4 is strongly upregulated in activated fibroblasts,^18^ significantly higher numbers of Nox4 mRNA‐expressing cells (0.73%, *p* < 0.001) and an abundance of densely stained clusters (denoting the presence of multiple transcripts/cell) were observed in PCa, particularly within the peritumoral stroma (Supporting Information, Fig. S2).

**Figure 1:**
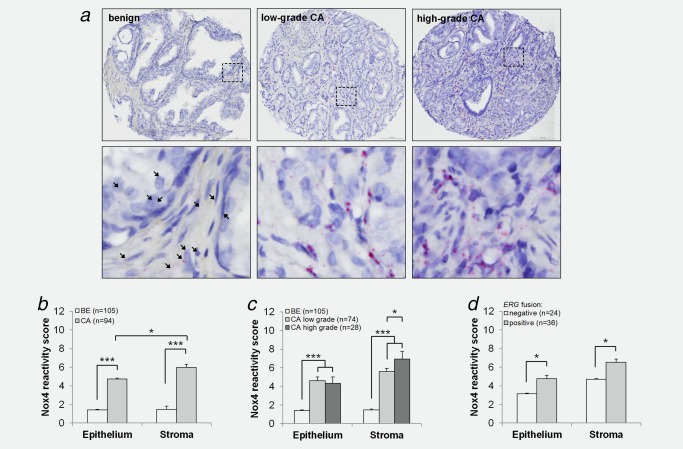
Nox4 expression is elevated in clinical PCa. PCa TMA stained via ISH for Nox4 transcript. (*a*) Representative images of benign (BE) or cancer (CA) foci are shown. Low‐grade and high‐grade CA was defined as predominantly Gleason pattern 3 or lower (Gleason score ≤3 + 4) and predominantly Gleason pattern 4 or higher (Gleason score ≥4 + 3), respectively. Single and multiple Nox4 mRNA transcripts appear as single red dots or clusters, respectively. Boxed regions (*top panel*) are enlarged (*lower panel*). Arrowheads demarcate weak Nox4 staining in benign tissue. Original magnification: 20× (*top panel*) and 40× (*lower panel*). (*b*–*d*) Quick‐score quantification of (*a*) as described in Materials and Methods. (*b*) Comparison of benign (BE) *versus* cancer (CA), (*c*) stratification of cancer cases into low or high Gleason grading as defined in (*a*) or (*d*) stratification of cancer cases according to *ERG*‐fusion status. (*b*–*d*) Data represent mean + SEM. Number of patients analyzed is indicated (*n*). Statistical significance is shown (*p < 0.05; ***p < 0.001).

Similar results were observed on the PCa TMA (Fig. 1 *a*) with Nox4 mRNA levels significantly elevated in both tumor epithelial and peritumoral stromal cells in PCa compared to patient‐matched benign regions. Notably, Nox4 mRNA levels were highest in the tumor‐associated stroma with ∼30% to >60% of cells positive for Nox4 staining (Fig. 1 *ab* and Supporting Information, Fig. S3). We observed no correlation of Nox4 expression (either stromal or epithelial) with Gleason score or tumor stage, although this may have been due to insufficient numbers of patients, particularly those with high grade PCa. To partially overcome this issue, patient samples were stratified into low‐ and high‐grade PCa (defined as predominantly Gleason pattern ≤3 + 4 and ≥4 + 3, respectively). While epithelial Nox4 mRNA levels did not differ between low‐ and high‐grade PCa, stromal Nox4 mRNA levels were significantly higher in high‐grade than low‐grade PCa (Fig. 1 *c* and Supporting Information, Fig. S3). In addition, stromal and epithelial Nox4 mRNA levels were significantly increased in *ERG‐*fusion‐positive PCa compared to *ERG*‐fusion‐negative tumors (Fig. 1 *d* and Supporting Information, Fig. S3). Similar increases in Nox4 expression in the PCa‐associated stroma compared to benign stroma were observed in several publicly available transcriptome profiling studies (Supporting Information, Fig. S4). To further validate these findings in independent cohorts, we scrutinized the Oncomine database and observed increased Nox4 mRNA levels not only in several PCa studies but also in other tumor types, particularly those with a prominent stromal component such as breast, gastric and pancreatic cancer (Supporting Information, Fig. S5).

### Elevated stromal Nox4 is spatially associated with epithelial TGFβ in clinical PCa

**Figure 2:**
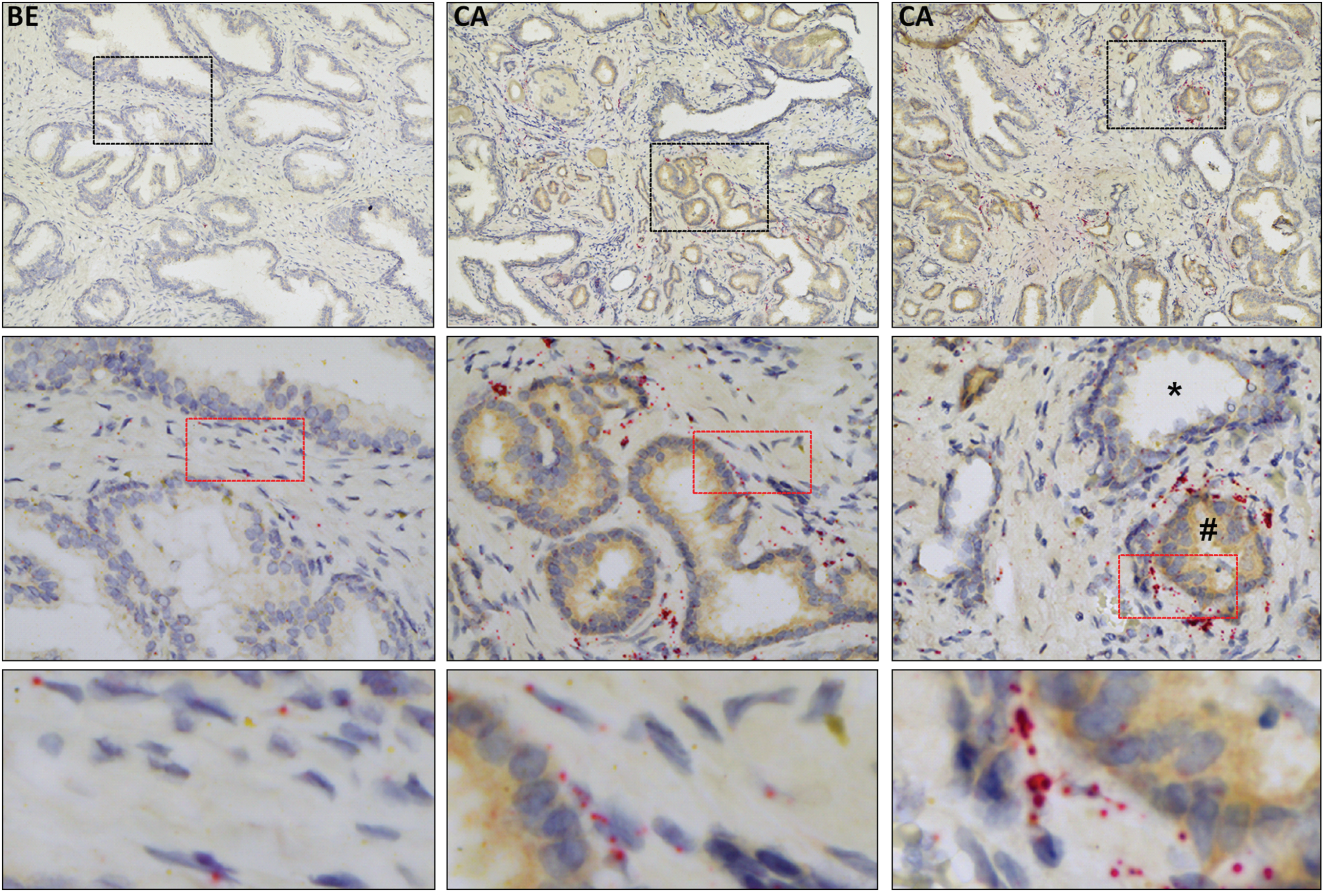
Elevated Nox4 mRNA in the PCa‐associated stroma is associated with epithelial TGFβ expression. Dual Nox4 *in situ* hybridization and TGFβ immunohistochemistry (pan TGFβ antibody) on radical prostatectomy prostate tissue from PCa patients. Two cancer (CA) and one benign (BE) adjacent areas are shown. Single and multiple Nox4 mRNA transcripts appear as single red dots or clusters, respectively. Yellow/brown chromogen staining for TGFβ. Original magnification: top panel 20×, middle and bottom panels 40×. Images are representative of 3 independent experiments using tissue isolated from different patients. Middle right panel: *, benign gland expressing very low levels of TGFβ and little stromal Nox4 with adjacent TGFβ‐positive tumor cells (#) surrounded by abundant Nox4 expression in the tumor‐associated stroma. Black and red boxed regions are enlarged in the middle and lower panels, respectively. Images of parallel‐stained negative controls are depicted in Supporting Information, Figure S6.

TGFβ ligands are potent inducers of Nox4 expression, fibroblast activation *in vitro* and *in vivo* and are abundantly expressed by epithelial cells in premalignant prostatic intraepithelial neoplasia (PIN) lesions and tumor cells in PCa.^8^ We therefore performed dual Nox4 ISH and IHC for TGFβ1/2/3 ligands on the same PCa tissue section to determine their spatial expression in PCa. Similar to single protocols for Nox4 ISH or TGFβ IHC, dual staining yielded a strong increase in Nox4 mRNA levels in the PCa‐associated stroma and a strong increase in epithelial cell TGFβ immunopositivity in cancerous regions compared to adjacent benign areas (Fig. 2 and Supporting Information, Fig. S6). Moreover, stromal areas with clusters of intense Nox4 staining were localized adjacent to tumor foci with abundant TGFβ staining (Fig. 2 and Supporting Information, Fig. S6). Together these data are strongly suggestive of a pathophysiological relationship between elevated tumor cell‐derived TGFβ and elevated Nox4 expression in the tumor‐associated stroma of clinical PCa.

### Nox4 but not Nox1 is the critical Nox isoform during TGFβ1‐mediated fibroblast activation

**Figure 3:**
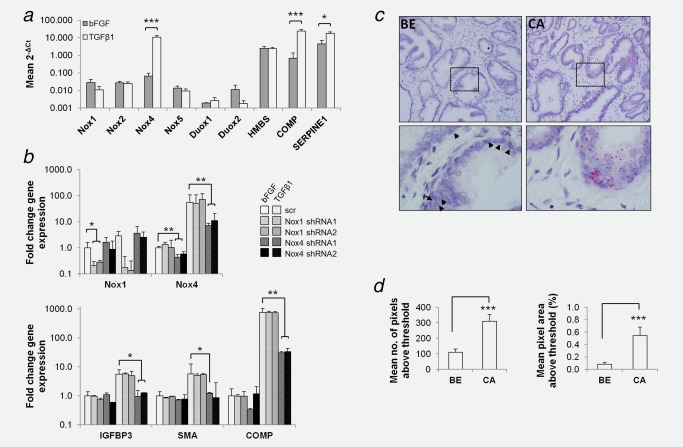
Nox4 but not Nox1 is critical for TGFβ1‐mediated fibroblast activation. (*a*) Primary human prostate fibroblasts incubated with bFGF or TGFβ1 as indicated for 48 hr before qPCR analysis of Nox family members relative to the housekeeping gene TBP. The direct TGFβ1 target genes SERPINE1 and COMP served as positive controls. The nonresponsive gene HMBS served as negative control. (*b*) Lentiviral‐mediated silencing of Nox1 or Nox4 in primary human prostate fibroblasts transduced with 2 different Nox1 or Nox4‐specific shRNAs (1 and 2) and subsequently treated with bFGF or TGFβ1 for 48 hr before qPCR of Nox1 and Nox4 expression (*top)*, or the indicated CAF marker (*bottom)* relative to the housekeeping gene TBP. (*c*,*d*) Nox1 RNA *in situ* hybridization on radical prostatectomy prostate tissue specimens from PCa patients. Benign (BE) and adjacent cancer (CA) areas are shown. Single and multiple Nox4 mRNA transcripts appear as single red dots or clusters, respectively. Boxed regions (*top panel*) are enlarged (*lower panel*). Arrowheads demarcate weak epithelial Nox1 mRNA levels in benign tissue. (*d*) ImageJ quantification of Nox1 *in situ* hybridization images as in (*c*) using threshold settings described in Materials and Methods. (*a*,*b*) Data represent mean + SEM of four independent experiments using primary fibroblasts isolated from different donors. (*c*) Images are representative of 3 independent experiments using tissue from different patients. (*d*) Data represent mean + SEM from 10 fields of view each (40× magnification) from CA and BE adjacent regions of 3 different patient specimens. (*a*, *b*, *d*) Statistical significance is shown (*p < 0.05; **p < 0.01, ***p < 0.001).

Subsequent efforts focused on addressing the functional consequences of pharmacologically targeting stromal Nox4 with respect to PCa‐promoting pathways. We therefore employed a well‐characterized dual Nox1/Nox4 inhibitor (GKT137831),^30^ which currently represents the only inhibitor that targets Nox4. As GKT137831 also displays high affinity for Nox1, we first established whether Nox1 is also functionally relevant with respect to prostate stromal activation. Basal expression levels of all Nox/Duox isoforms in primary human prostate fibroblasts were very low and, with the exception of Nox4, not significantly altered upon TGFβ1‐mediated activation^18^ (Fig. 3 *a*). The direct TGFβ1‐target genes serpin family E member 1 (SERPINE1) and cartilage oligomeric matrix protein (COMP) and nonresponsive housekeeping gene hydroxymethylbilane synthase (HMBS) served as positive and negative controls, respectively. Moreover, Nox1 mRNA could not be detected via ISH in primary prostate fibroblasts either under nonactivating (bFGF) or activated (TGFβ1) conditions (Supporting Information, Fig. S1B). In addition, Nox4‐ but not Nox1‐specific shRNA attenuated TGFβ1‐mediated induction of CAF markers FAP, SMA, IGFBP3 and COMP (Fig. 3 *b* and data not shown). Moreover, while Nox1 mRNA was sparsely detected in epithelial cells of benign prostate tissue (0.08% of cells in benign areas showed 1–2 mRNA transcript signals) and significantly increased in tumor epithelial cells of adjacent cancer foci (0.55%, *p <* 0.0001; Fig. 3 *cd*), Nox1 mRNA was not detectable in either the benign or tumor‐associated stroma of prostate tissue specimens (Fig. 3 *c*). Collectively, these data indicate that Nox4 is the critical Nox isoform during TGFβ1‐mediated fibroblast activation to a CAF‐like phenotype and that Nox1 plays at the most only a very minor role.

### Pharmacological inhibition of Nox4 attenuates TGFβ1‐mediated prostate fibroblast activation

**Figure 4:**
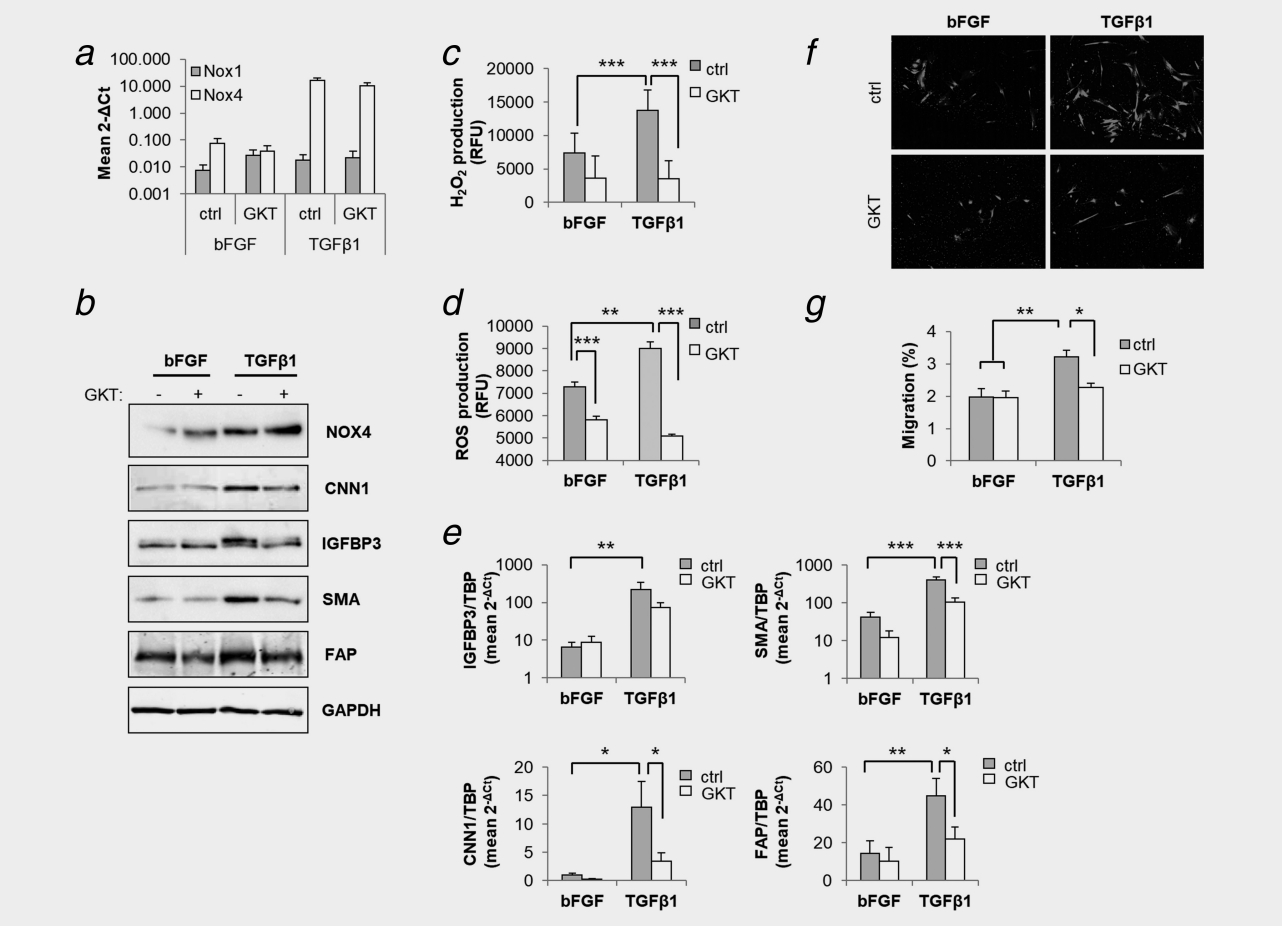
GKT137831 attenuates TGFβ1‐mediated fibroblast activation. Primary prostate human fibroblasts were treated with bFGF or TGFβ1 in the presence of 30 μM GKT137831 (GKT) or DMSO equivalent (control, ctrl) for 48 hr before (*a*) qPCR of Nox1 or Nox4 expression relative to the housekeeping gene TBP, (*b*) Western blotting of total cell extracts using the indicated antibodies and GAPDH as loading control, (*c*) determination of extracellular H_2_O_2_ levels via Amplex Red assay, (*d*) quantification of intracellular ROS via CM‐H_2_DCFDA staining, (*e*) qPCR determination of the indicated CAF markers, and (*f*,*g*) analysis of migration using Boyden chamber transwell assays. (*a*,*c*–*e*,*g*) Data represent mean + SEM of at least four independent experiments using primary fibroblasts isolated from different donors. Statistical significance is shown (**p < 0.01; ***p < 0.001). (*b*) GAPDH served as loading control. (*b*,*f*) Images are representative of 3 independent experiments using cells isolated from different donors. (*c*,*d*) RFU, relative fluorescent units. Statistical significance is shown (*p < 0.05; **p < 0.01; ***p < 0.001).

To investigate the therapeutic potential of pharmacological Nox4 inhibition with respect to attenuating stromal activation, GKT137831 was applied to primary human prostate fibroblasts. GKT137831 had no significant effect on Nox1/Nox4 mRNA levels in basal or TGFβ1‐treated prostate fibroblasts although we noted a nonsignificant trend toward increased Nox4 protein levels (Fig. 4 *ab* and Supporting Information, Figs. S7 and S8B). In addition, GKT137831 had no significant effect on prostate fibroblast proliferation (Supporting Information, Fig. S8C). However, GKT137831 dose‐dependently decreased TGFβ1‐induced H_2_O_2_/ROS production in prostate fibroblasts (Fig. 4 *cd* and Supporting Information, Fig. S8D). In addition, GKT137831 significantly attenuated TGFβ1‐induced expression of CAF markers at both the mRNA and protein level (Fig. 4 *be* and Supporting Information, Fig. S9). Moreover, GKT137831 significantly attenuated the elevated migratory capacity of activated fibroblasts (Fig. 4 *fg*). Similar effects were observed upon shRNA‐mediated knockdown of Nox4 but not Nox1 (Fig. 3 *b* and data not shown). These data together with the aforementioned findings that Nox1 is expressed at low/non‐detectable levels in prostate fibroblasts (Fig. 3 and Supporting Information, Fig. S1B) strongly suggest that GKT137831 ablates TGFβ1‐induced fibroblast activation primarily by inhibiting the ROS‐producing activity of Nox4.

### Nox4 is required for pro‐proliferative stromal‐derived paracrine effects on AR^+^ PCa cells

**Figure 5:**
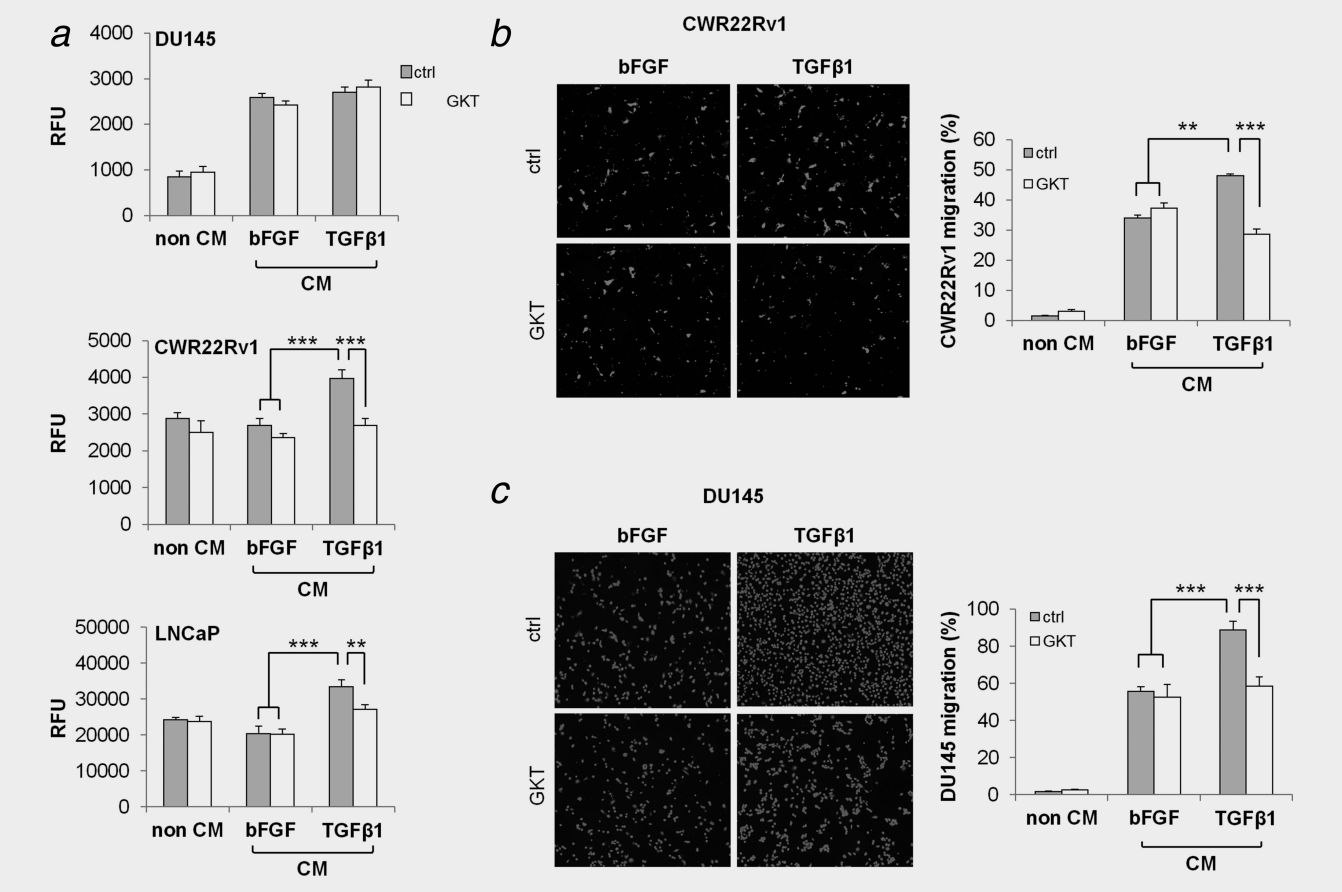
GKT137831 attenuates paracrine‐mediated induction of PCa cell proliferation and migration. Primary human prostate fibroblasts were treated with bFGF or TGFβ1 in the presence of GKT137831 (GKT) or DMSO equivalent (control, ctrl) for 48 hr. Cells were washed with serum‐free media and further incubated in serum‐free media in the presence of GKT137831 or DMSO equivalent for 48 hr before collection of conditioned media (CM). (*a*) Fibroblast CM or non‐CM (serum‐free DMEM containing 30 µM GKT137831 or DMSO equivalent incubated without fibroblasts) was applied to the indicated PCa cell line for 96 hr before analysis of proliferation via SybrGreen assay. (*b*,*c*) CM or non‐CM (serum‐free DMEM containing 30 µM GKT137831 or DMSO equivalent incubated without cells) was used as chemoattractant in the lower chamber of transwell migration assays for (*b*) CWR22Rv1 or (*c*) DU145 cells seeded in serum‐free media in transwell inserts and migrating cells analyzed (*b*) 24 hr or (*c*) 48 hr later. (*a*–*c*) Data represent mean + SEM of three independent experiments using CM harvested from primary fibroblasts isolated from different donors. Statistical significance is depicted (**p < 0.01; ***p < 0.001). Representative images are shown.

Remaining experiments focused on addressing the functional significance of stromal Nox4 with respect to PCa‐promoting processes. CAFs secrete a variety of mitogenic factors.^2^ Thus, conditioned media (CM) harvested from fibroblasts activated with TGFβ1 (or bFGF control) in the presence or absence of GKT137831 was applied to different PCa cell lines. Consistent with their low expression of Nox1 and Nox4 (Supporting Information, Fig. S10), the inhibitor alone (non‐CM) had no significant effect on the proliferation of CWR22Rv1, LNCaP (both androgen receptor positive, AR^+^) or DU145 (androgen receptor negative, AR^−^) cells (Fig. 5 *a*). CM from activated fibroblasts (neither in the presence or absence of GKT137831) had no significant effect on the proliferation of AR^‐^ DU145 cells compared to CM from nonactivated stromal cells (Fig. 5 *a*). In contrast, CM from TGFβ1‐activated fibroblasts but not nonactivated fibroblasts (bFGF treated) significantly increased the proliferation of AR^+^ LNCaP and CWR22Rv1 cells, an effect that was abrogated by GKT137831 (Fig. 5 *a*). Similarly, Nox4‐specific knockdown also attenuated stromal‐induced proliferation of LNCaP/CWR22Rv1 cells by TGFβ1‐activated fibroblasts (not shown).

### Nox4 is required for promigratory stromal‐derived paracrine effects on PCa cells

In addition to promoting tumor cell proliferation, CAFs also stimulate tumor cell migration, at least in part via their secretion of chemotaxins (e.g., CXCL12) and matrix metalloproteinases.^2^ We therefore analyzed PCa cell migration using stromal CM as chemoattractant harvested from fibroblasts activated with TGFβ1 (or bFGF control) in the presence or absence of GKT137831. CM from activated fibroblasts significantly enhanced migration of both AR^+^ and AR^−^ PCa cells compared to CM from nonactivated (bFGF‐treated) fibroblasts (Fig. 5 *bc*). Moreover, whilst CM from nonactivated fibroblasts treated with GKT137831 did not modulate the migration of DU145 or CWR22Rv1 cells relative to control, the promigratory response of both cell lines to CM from activated fibroblasts was significantly attenuated in the presence of GKT137831 (Fig. 5 *bc*). Similar effects on PCa cell migration were observed upon Nox4 knockdown (Supporting Information, Fig. S11).

### PCa cells induce fibroblast activation via stromal TGFβ receptor and Nox4 signaling

**Figure 6:**
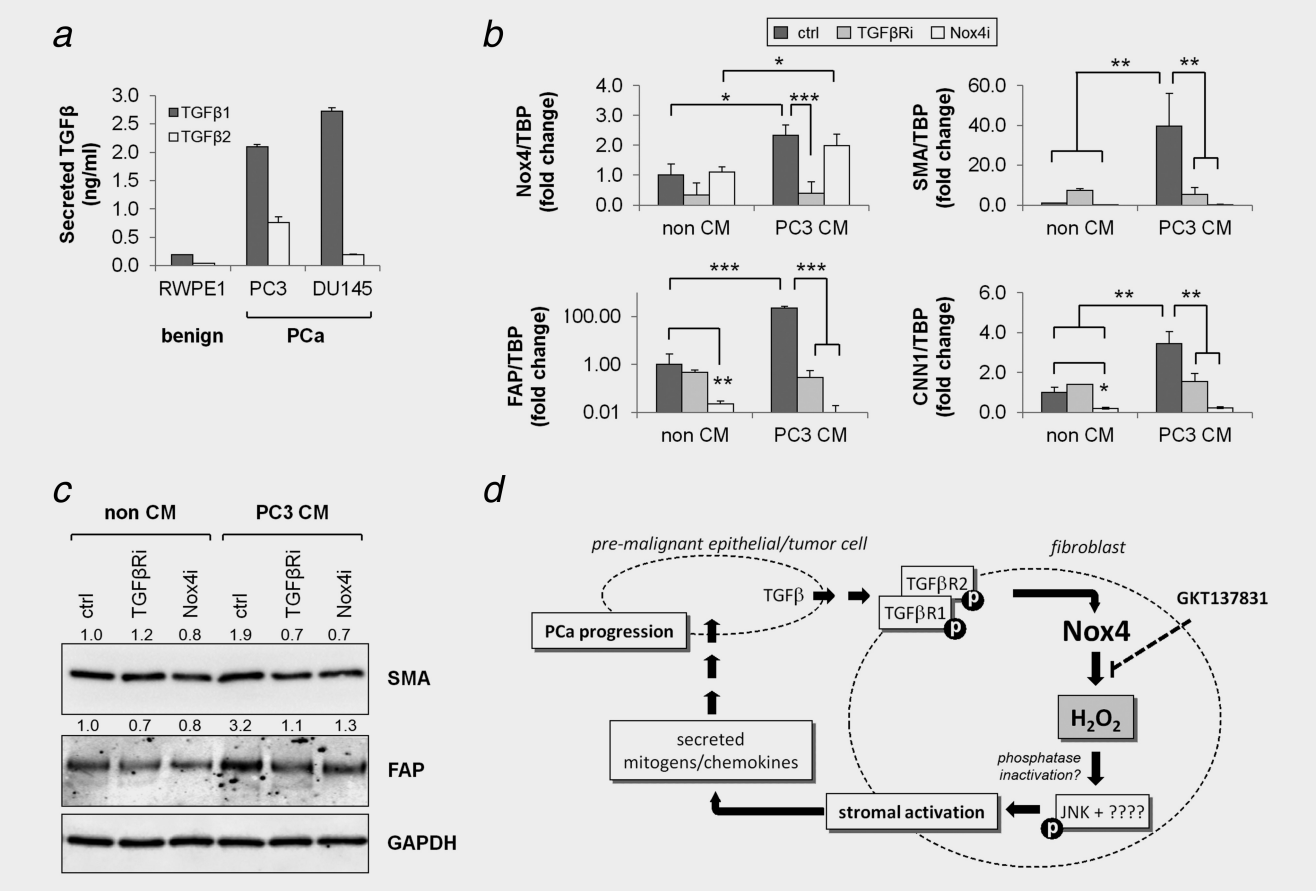
PCa cell‐derived TGFβ induces fibroblast activation in a paracrine manner dependent on stromal Nox4. (*a*) ELISA quantification of TGFβ1 or TGFβ2 in serum‐free conditioned media (CM) harvested from the indicated prostate epithelial cell line after 72 hr and normalized against non‐CM (serum‐free media incubated without cells). Data represent mean concentration + SEM from duplicate measurements of 3 independent experiments using 3 different CM isolates. qPCR (*b*) or Western blotting (*c*) of prostate fibroblasts pretreated with 1 μM TGFβR inhibitor SB431452 (TGFβRi), 30 μM Nox4 inhibitor GKT137831 (Nox4i) or vehicle equivalent (control, ctrl) in serum‐free DMEM before addition of PC3 PCa cell CM or non‐CM control (serum‐free media incubated without cells). (*b*) Data represent mean fold change + SEM in gene expression relative to the housekeeping gene TBP from three independent experiments using primary fibroblasts isolated from different donors. Statistical significance is shown (*p < 0.05; **p < 0.01; ***p < 0.001). (*c*) Images are representative of 3 independent experiments using cells isolated from different donors. (*d*) Schematic overview of the proposed mechanism underlying the protumorigenic actions of stromal Nox4. Elevated secretion of TGFβ1 by premalignant epithelial cells in prostatic intraepithelial neoplasm (PIN) lesions and PCa tumor cells activates TGFβ signaling in adjacent stromal fibroblasts and induces Nox4‐derived H_2_O_2_ production, which subsequently promote JNK phosphorylation (and perhaps other as yet unidentified kinases) (18), most likely via oxidative inactivation of JNK‐phosphatase(s), resulting in fibroblast activation to the CAF phenotype. In turn, activated fibroblast‐derived paracrine‐acting factors promote tumor cell proliferation and migration and thus PCa development/progression. As a central mediator in this cascade of dysregulated stromal–epithelial interactions, abrogating the ROS‐producing activity of Nox4 (e.g., via GKT137831) represents a promising therapeutic strategy for PCa.

Experiments thus far employed recombinant TGFβ1 to mimic elevated TGFβ secretion by epithelial cells in PIN/PCa for induction of stromal activation. Thus, we next sought to determine whether GKT137831/Nox4 inhibition could also abrogate stromal activation in the more physiological setting of the complex milieu secreted by PCa cells. In agreement with its benign status the prostate epithelial cell line RWPE1 secreted only very low levels of TGFβ1 and TGFβ2, whereas DU145 and PC3 PCa cell lines secreted significantly higher levels of TGFβ2 and even greater levels of TGFβ1 (>2 ng/ml; Fig. 6 *a*). Consistently, CM from PC3 cells induced stromal activation of primary prostate human fibroblasts as demonstrated by the induction of Nox4, SMA, CNN1 and FAP at both the mRNA and protein level (Fig. 6 *bc*). Moreover, fibroblast activation by PC3 CM was significantly ablated when fibroblasts were treated with the TGFβ receptor inhibitor SB431542 or the Nox1/Nox4 inhibitor GKT137831 prior to the addition of PC3 CM (Fig. 6 *bc*). These data indicate that PCa cell‐derived TGFβ can activate prostate fibroblasts via stromal TGFβ receptor and Nox4‐derived ROS signaling and that GKT137831 can attenuate PCa cell‐driven stromal activation (summarized in Fig. 6 *d*).

## Discussion

Our previous observations that elevated Nox4 expression is associated with aggressive PCa as defined by biochemical relapse together with experimental findings that Nox4 knockdown is sufficient to abrogate TGFβ1‐mediated activation of prostate fibroblasts, prompted us to further characterize the role of prostatic Nox4 and investigate the potential utility of pharmacological inhibition of Nox4 as a therapeutic strategy for PCa.

We demonstrate for the first time that the tumor‐associated PCa stroma is the predominant source of elevated Nox4 in clinical PCa. Genes encoding the ETS family of transcription factors (including ERG) are frequently rearranged in PCa with ERG overexpression shown to drive invasion and metastasis.^31^ Thus, the significant increase in stromal Nox4 expression in high‐grade and *ERG‐*fusion‐positive PCa *versus* low‐grade and *ERG‐*fusion‐negative PCa, respectively, together with our previous finding that total Nox4 mRNA levels correlate with biochemical relapse^19^, ^20^ implicate an active role of stromal Nox4 in PCa progression. This hypothesis is supported by the finding that total Nox4 mRNA levels were significantly higher in lethal *versus* nonlethal *ERG*‐positive PCa.^19^, ^20^

The molecular mechanisms driving fibroblast activation to the CAF phenotype remain poorly defined however several cytokines and growth factors (e.g., interleukins‐6, −8 and TGFβ) have been implicated.^32^ Taken together with reports that Nox4 is a direct transcriptional target of TGFβ/Smad signaling,^33^ the striking spatial association between expression of stromal Nox4 and TGFβ in tumor cells further implicate epithelial‐derived TGFβ as a crucial inducer of reactive stroma and underlying cause of elevated Nox4 expression in clinical PCa.

*In vitro* studies herein illustrate the physiological relevance of elevated stromal Nox4 in PCa and demonstrate its role as a central mediator of (i) reciprocal epithelial‐stromal cell crosstalk, (ii) fibroblast activation and (iii) stromal‐driven tumor (cell)‐promoting hallmarks (summarized in Fig. 6 *d*). As we previously demonstrated, mechanistically Nox4 mediates these effects by as yet poorly understood ROS‐dependent processes, for example, Nox4‐derived ROS‐dependent phosphorylation of JNK is essential for TGFβ1‐driven prostate fibroblast activation.^18^ In addition, Nox4‐derived ROS have been shown to regulate migration via oxidation of proteins involved in integrin activation and cytoskeletal remodeling.^34^ This may at least in part explain our observation that the elevated migratory capacity of activated fibroblasts is dependent on Nox4 activity, a pertinent finding given that migrating fibroblasts at the leading edge of the tumor invasive front remodel the ECM and thereby facilitate cancer cell migration and invasion.^35^

Nox4‐derived ROS are primarily detectable as H_2_O_2_, which unlike superoxide is membrane‐permeable and has a longer half‐life rendering it an ideal intracellular but also extracellular signaling mediator.^13^ Interestingly, TGFβ1‐mediated fibroblast activation requires both intra‐ and extracellular ROS as membrane‐permeable or ‐impermeable forms of antioxidant enzymes abrogate the induction of different sets of CAF markers by TGFβ1 (not shown). Such extracellular functions of ROS have been described previously, for example, TGFβ‐induced ROS activate latent TGFβ and cross‐link fibroblast‐derived ECM proteins.^36^, ^37^ Further studies are underway to identify the direct oxidative targets of Nox4‐derived ROS during TGFβ1‐mediated fibroblast activation.

CAFs comprise a highly heterogeneous cell population with different CAF subtypes shown to exert either tumor‐promoting or tumor‐inhibitory effects in PCa.^9^ *In vitro* data herein demonstrating that Nox4 plays a central role in paracrine‐mediated pro‐proliferative and promigratory effects of activated fibroblasts on PCa cells strongly suggest that Nox4 inhibition will exert antitumorigenic effects. Moreover, as Nox4 positivity is not observed homogeneously throughout the tumor microenvironment, these observations lead us to hypothesize that high Nox4 expression is not a general reactive stromal marker but may reflect/define a particular onco‐supportive CAF subtype.

While the precise soluble effectors of Nox4‐derived ROS remain to be identified, the differential effects with respect to proliferation and migration of AR^+^ *versus* AR^−^ PCa cells clearly indicates the involvement of different pathways. For example, the stimulation of both AR^+^ and AR^‐^ PCa cell migration by CM of activated fibroblasts with intact stromal Nox4 signaling suggests this is occurring in a manner independent of epithelial AR. This may be attributed to the well‐documented secretion of ECM remodeling enzymes and chemotactic factors by activated fibroblasts.^5^ In contrast, the increase in proliferation of AR^+^ but not AR^−^ cell lines upon exposure to CM from activated but not nonactivated fibroblasts suggest potential crosstalk with epithelial AR, a hypothesis consistent with reports that TGFβ1‐activated stromal cells differentially express several androgen‐metabolizing enzymes.^38^ Notably, proliferation of the AR^‐^ DU145 cells was equally stimulated by the conditioned media of both nonactivated and activated fibroblasts and thus plausibly by the same stromal‐derived factor(s). These observations reflect the complex heterogeneity of clinical PCa and are the subject of ongoing studies. As Nox4 mRNA levels are also elevated in nonprostate tumors (Supporting Information, Fig. S3A), findings herein are also expected to be relevant to other tumor types with a prominent stromal component (e.g., pancreatic ductal carcinoma and cancer of the breast, lung and liver) where a role of Nox4 has been implicated.^39^, ^40^, ^41^

Elevated ROS and NADPH oxidases have long been implicated in playing a role in prostate carcinogenesis. Several prominent clinical trials however employing dietary antioxidants yielded disappointing anticancer results.^42^, ^43^, ^44^ Thus, current consensus is that rather than attempting to neutralize ROS, treatment strategies aimed at reducing ROS production might represent an effective alternative approach.^45^ In this respect, GKT137831 is a pyrazolopyridine dione derivative and first in‐class potent and orally bioavailable dual Nox1/Nox4 inhibitor that displays excellent pharmacological and safety profiles.^30^ Consistent with the many molecular and cellular parallels between the desmoplastic stromal response in solid tumors and perturbed wound healing process in fibrosis,^46^ a TGFβ1‐Nox4 signaling axis has emerged as a common denominator in the activation of mesenchymal cells from numerous tissues with elevated Nox4 expression implicated in the pathophysiology of various fibrotic conditions.^47^ Consequently, GKT137831 is currently undergoing clinical trials following antifibrogenic effects in *in vitro* and *in vivo* experimental models of idiopathic pulmonary fibrosis, diabetic nephropathy, hepatic fibrosis and retinal inflammation.^30^, ^48^, ^49^, ^50^ Data herein indicate that the potential application of GKT137831 as a cancer therapeutic particularly for PCa should be evaluated.

In summary, we demonstrate that Nox4 inhibition via genetic or pharmacological strategies attenuates prostate fibroblast activation (induced either by PCa cell‐derived or recombinant TGFβ) and abrogates downstream paracrine‐mediated protumorigenic effects on PCa cells. Given the spatial association of abundant stromal Nox4 expression with TGFβ‐expressing tumor foci in clinical PCa, these findings collectively provide a strong mechanistic rationale for therapeutic inhibition of Nox4 as a stromal‐targeted approach to complement current treatment strategies for PCa and restore dysregulated reciprocal stromal–epithelial interactions.

## Supporting information

Supporting Information Figures

Supporting Information Table 1
