# Supplementary material for: Inhibition of Nox4‐dependent ROS signaling attenuates prostate fibroblast activation and abrogates stromal‐mediated protumorigenic interactions
Source: Int J Cancer. 2018 Mar 1;143(2):383–95. doi: 10.1002/ijc.31316 (PMC6067067; doi:10.1002/ijc.31316)
Supplement: Supplementary file 1 — Supporting Information Figures [file IJC-143-383-s001.pdf]

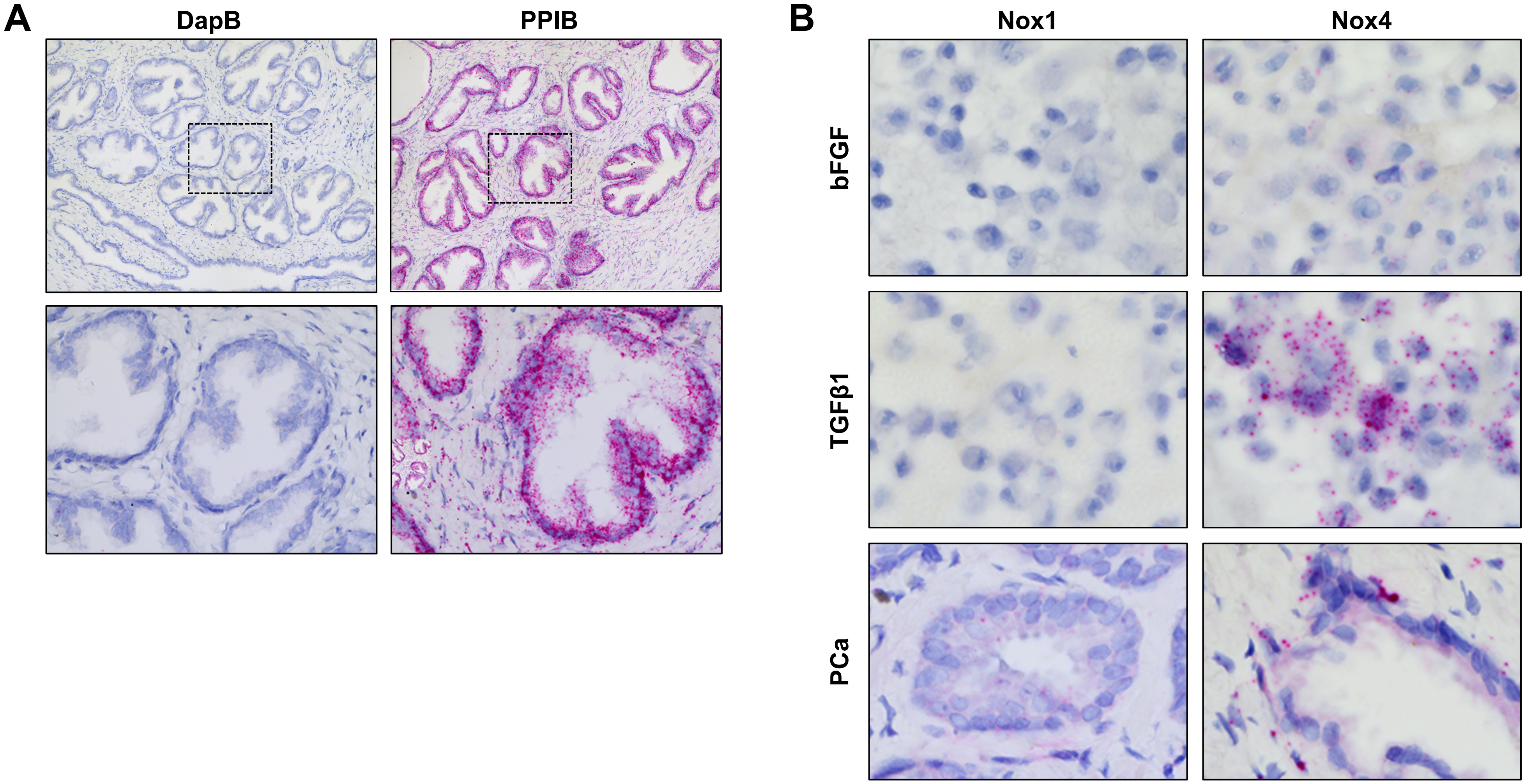

Supplemental Fig. S1 Specificity of *in situ* hybridization on prostate tissue sections and evaluation of Nox1/Nox4 mRNA levels in primary prostate fibroblasts

(A) RNA in situ hybridization for PPIB (positive) and DapB (negative) control probes on prostate tissue from patients undergoing radical prostatectomy for PCa. These control probes were run in parallel for all RNA *in situ* hybridization experiments to verify tissue integrity and assay specificity. Original magnification: top panel 20x, bottom panel 40x. **(B)** Nox1 or Nox4 in situ hybridization of formalin fixed paraffin embedded cell clots of primary prostate fibroblasts treated for 48h with bFGF or TGFβ1 as indicated. PCa tissue was stained in parallel as positive control. Original magnification 40x.

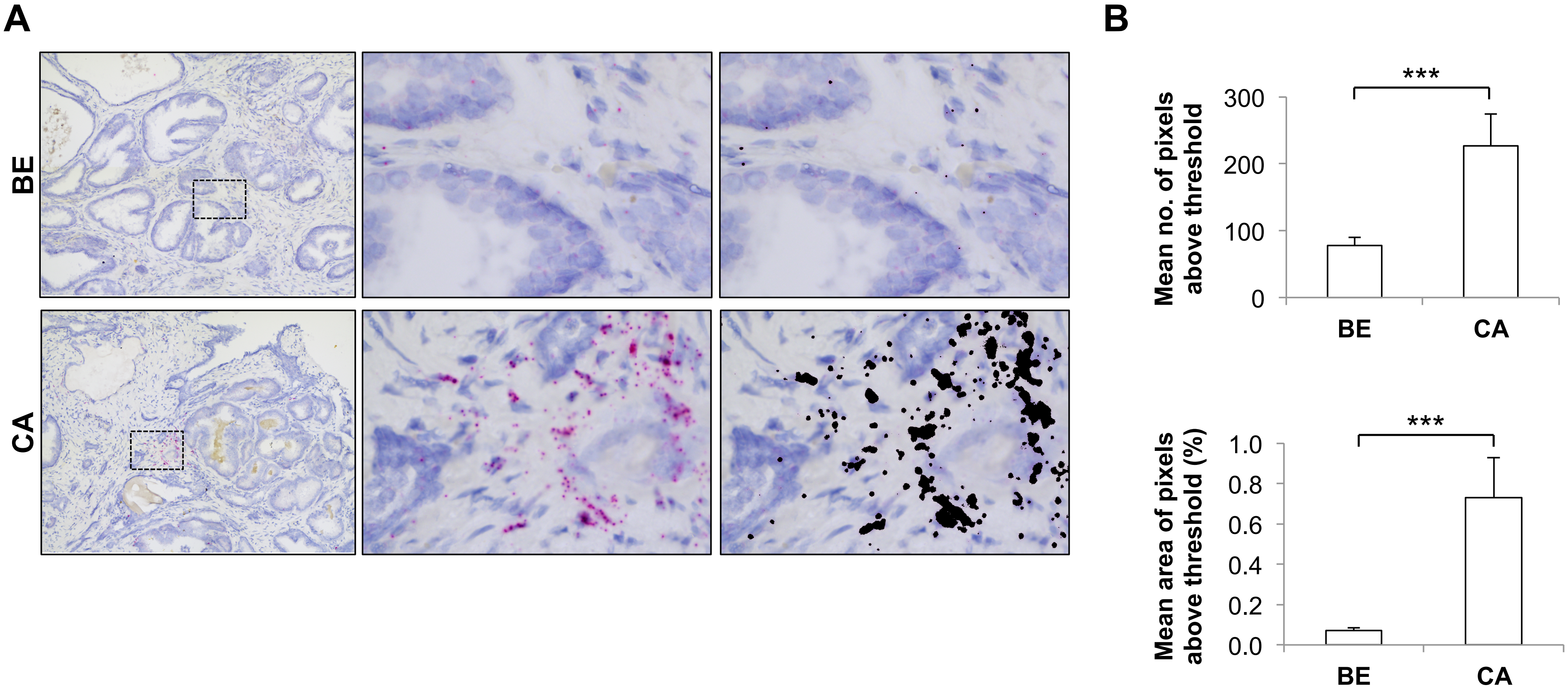

Supplemental Fig. S2 Nox4 expression is elevated in PCa

(A) Nox4 RNA *in situ* hybridization on radical prostatectomy prostate tissue specimens from PCa patients. Benign (BE) and adjacent cancer (CA) areas are shown. Single and multiple Nox4 mRNA transcripts appear as single red dots or clusters, respectively. Boxed regions (*left panel*) are enlarged (*middle panel*). ImageJ analysis of the same images with pixels above the threshold settings described in materials and methods colored black (*right panel*). **(B)** ImageJ quantification of images as depicted in (A). Data represent mean + SEM from 10 fields of view each (40x magnification) from CA and BE adjacent regions of 3 different patient specimens. Statistical significance is shown (***, P <0.001).

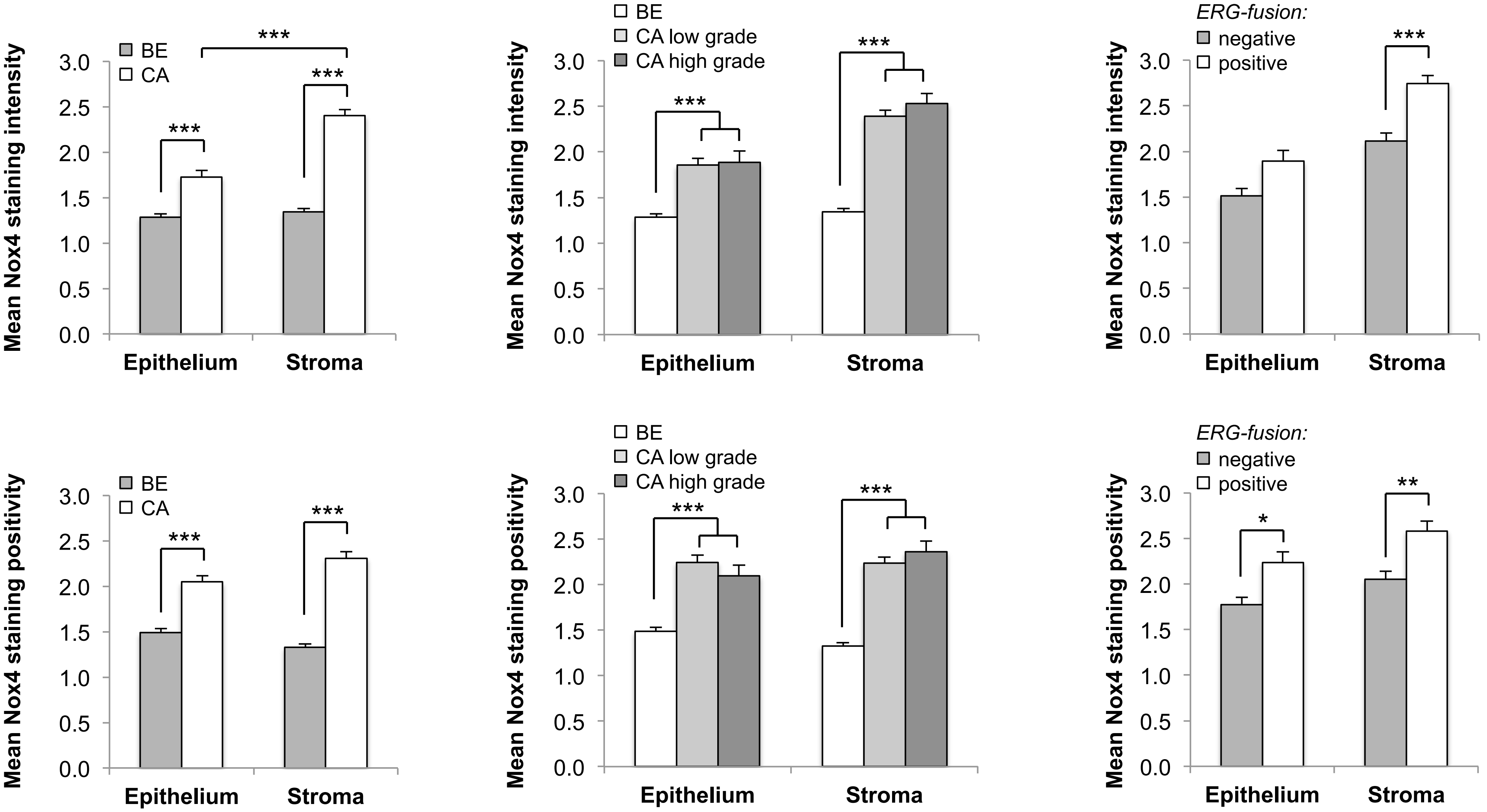

Supplemental Fig. S3 Comparison of Nox4 staining intensity *vs.* frequency of positive staining in human prostate cancer.

Separate analysis of Nox4 staining intensity (*top panels)* and proportion of Nox4 positively stained cells *(lower panels)* via *in situ* hybridization of PCa tissue microarrays as described in materials and methods. Cases were stratified into: LEFT, benign (BE) *vs.* cancer (CA); MIDDLE, low grade and high grade CA defined as predominantly Gleason pattern 3 or lower (Gleason score ≤3+4) and predominantly Gleason pattern 4 or higher (Gleason score ≥4+3), respectively and RIGHT, cancer cases according to *ERG*-fusion status. Data represent mean + SEM. Statistical significance is shown (*, $P < 0.05$; **, $P<0.01$; ***, $P < 0.001$).

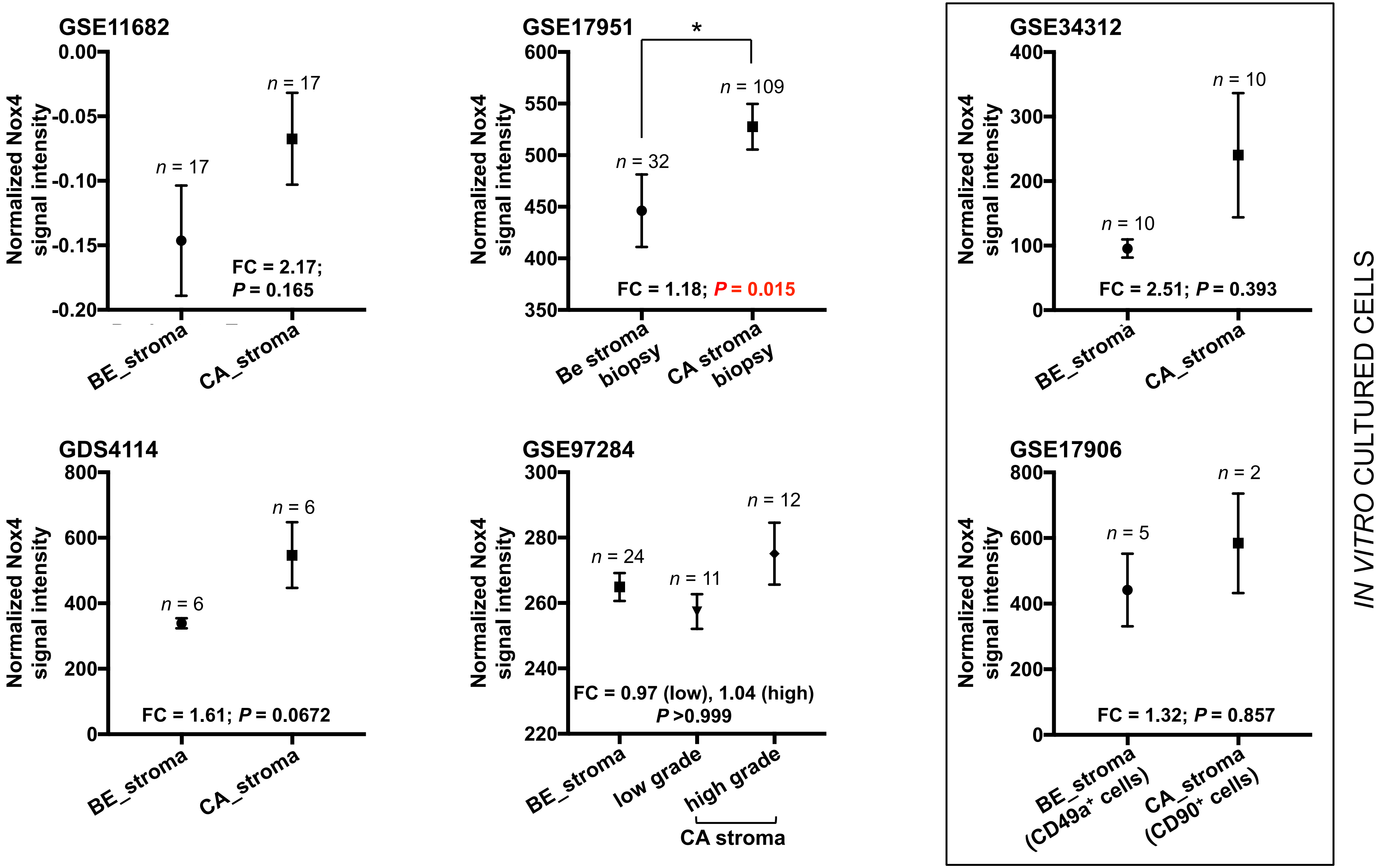

Supplemental Fig. S4 **Up-regulation stromal Nox4 mRNA levels in independent prostate cancer specimens**

Nox4 expression in benign (BE) or cancer (CA) stroma/stromal cells from prostate tissues as determined by GEO2R analysis of the indicated GEO dataset (GSE/GDE identifier) at https://www.ncbi.nlm.nih.gov/gds. Fold change in Nox4 gene expression (FC) in CA relative to BE samples is shown as well as *P* values (*P*). Statistical significance is shown (*, $P < 0.05$). Sample numbers are indicated (*n*).

A

Disease	Cancer *vs.* benign	
	Up	Down
Bladder cancer		
Brain and CNS cancer	4 (26)	**1** (26)
Breast cancer	**5** (45)	
Cervical cancer		
Colorectal cancer	**9** (33)	
Esophageal cancer	2 (9)	
Gastric cancer	**5** (20)	
Head and neck cancer	6 (27)	
Kidney cancer		7 (20)
Leukemia		
Liver cancer	2 (13)	
Lung cancer	6 (24)	
Lymphoma		
Melanoma	1 (7)	1 (7)
Myeloma		
Other cancer		
Ovarian cancer	2 (12)	
Pancreatic cancer	**5** (10)	
Prostate cancer	**3** (14)	
Sarcoma		
**No. significant analyses**	**50**	**9**
**Total no. analyses**	**373**	

B

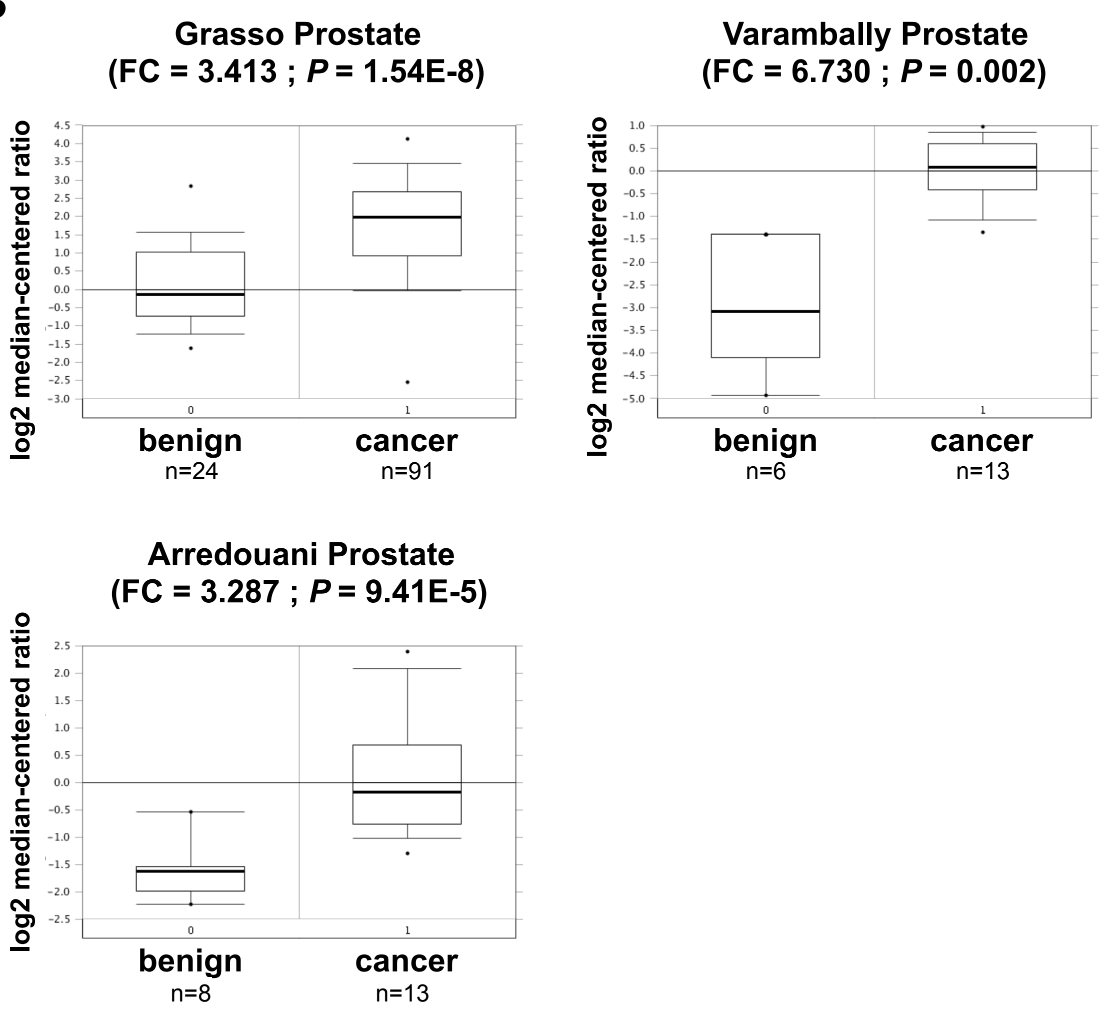

Supplemental Fig. S5 Nox4 expression is significantly increased in cancer

The Oncomine database was queried for datasets with a change in Nox4 mRNA levels of at least 1.5 fold (*P* <0.01) and top 10% of significantly regulated genes (gene rank percentile). **(A)** Disease summary of significantly scoring datasets. Values indicate the number of significant analyses for each disease type and numbers in parentheses indicate the total number of analyses. Dark and light grey shading denote the top 1% and top 5% gene rank percentile for the analyses within the cell, respectively. **(B)** Boxplot analysis of Nox4 mRNA levels in PCa-specific datasets. FC, fold change in Nox4 expression.

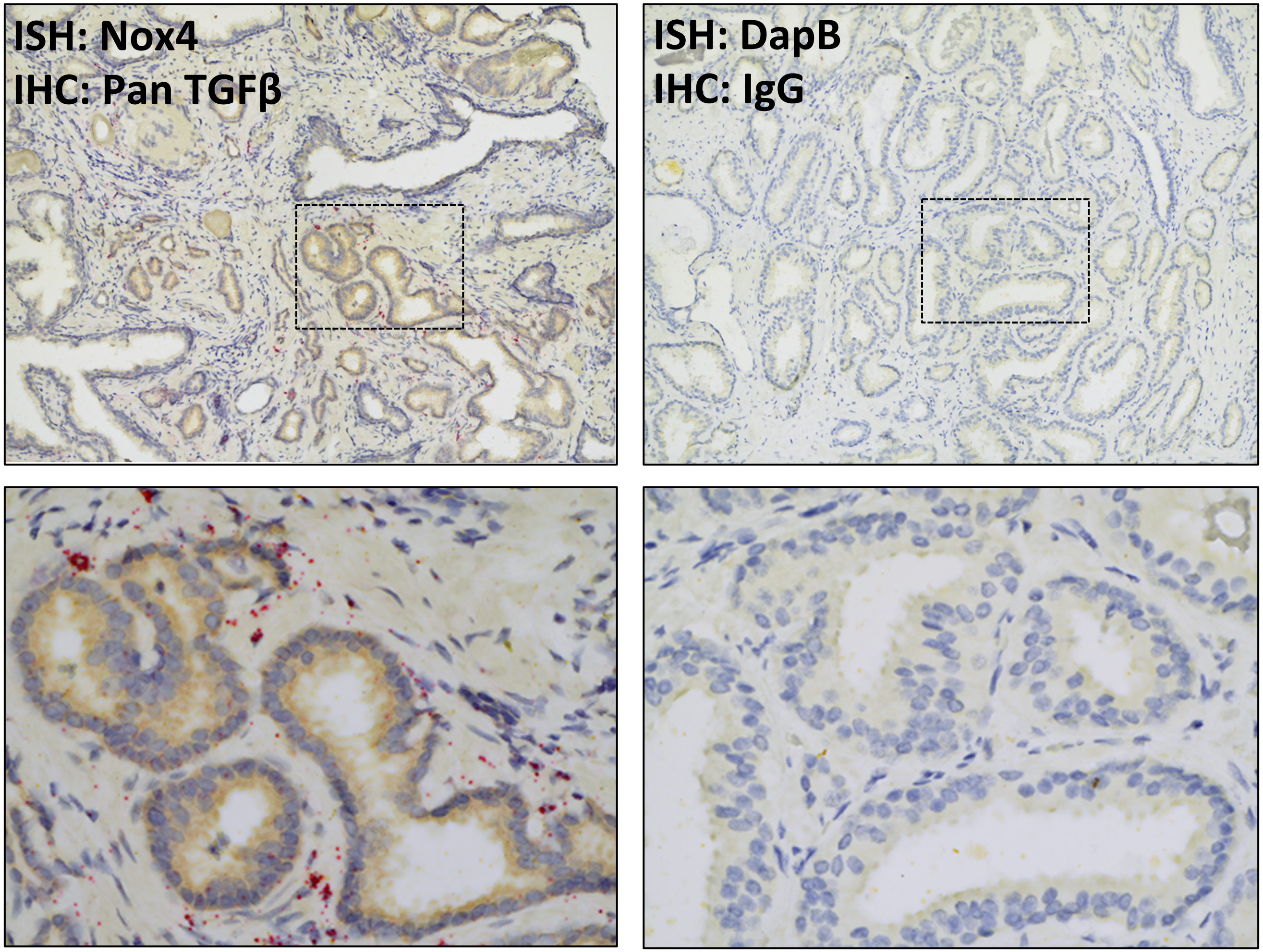

Supplemental Fig. S6 Specificity control of pan TGFβ immunostaining on PCa tissue

Dual Nox4 or DapB ISH with concurrent TGFβ (pan TGFβ antibody) or IgG control immunohistochemistry, respectively on radical prostatectomy prostate tissue from PCa patients. The experiment was performed in parallel with that depicted in Fig. 2. Cancer areas for both TGFβ and IgG are shown. Red staining denotes Nox4 ISH, yellow/brown chromogen staining for TGFβ immunohistochemistry. Original magnification: top panel 20x, bottom panel 40x. Images are representative of 3 independent experiments using tissue isolated from different patients.

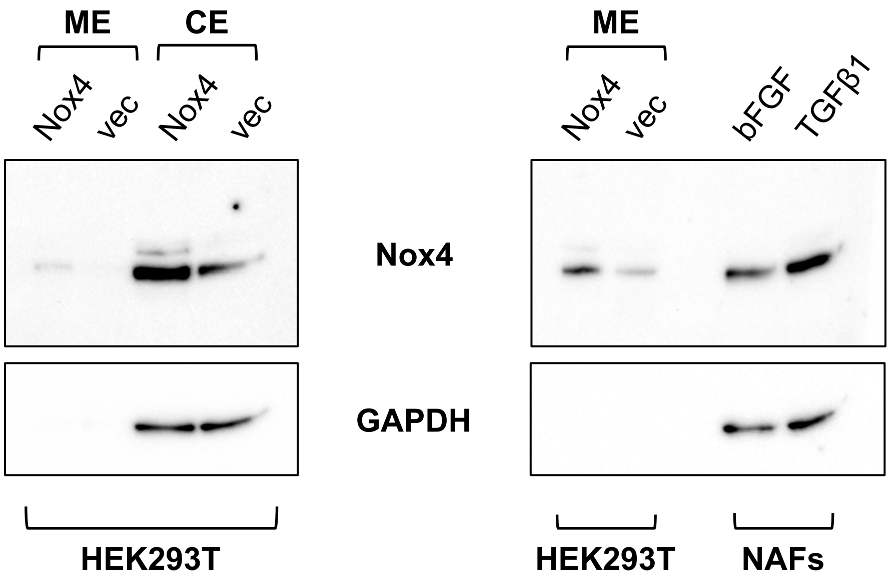

Supplemental Fig. S7 Validation of anti-Nox4 antibody specificity

Western blotting of total cell lysates from primary prostate human fibroblasts (NAFs) treated with bFGF or TGFβ1 for 48h as indicated using an anti-Nox4 antibody (Abcam Ab133303) or GAPDH as loading control. Membrane (ME) and cytosolic (CE) extracts prepared from HEK293T cells stably overexpressing Nox4 or empty vector control (vec) served as positive and negative control, respectively.

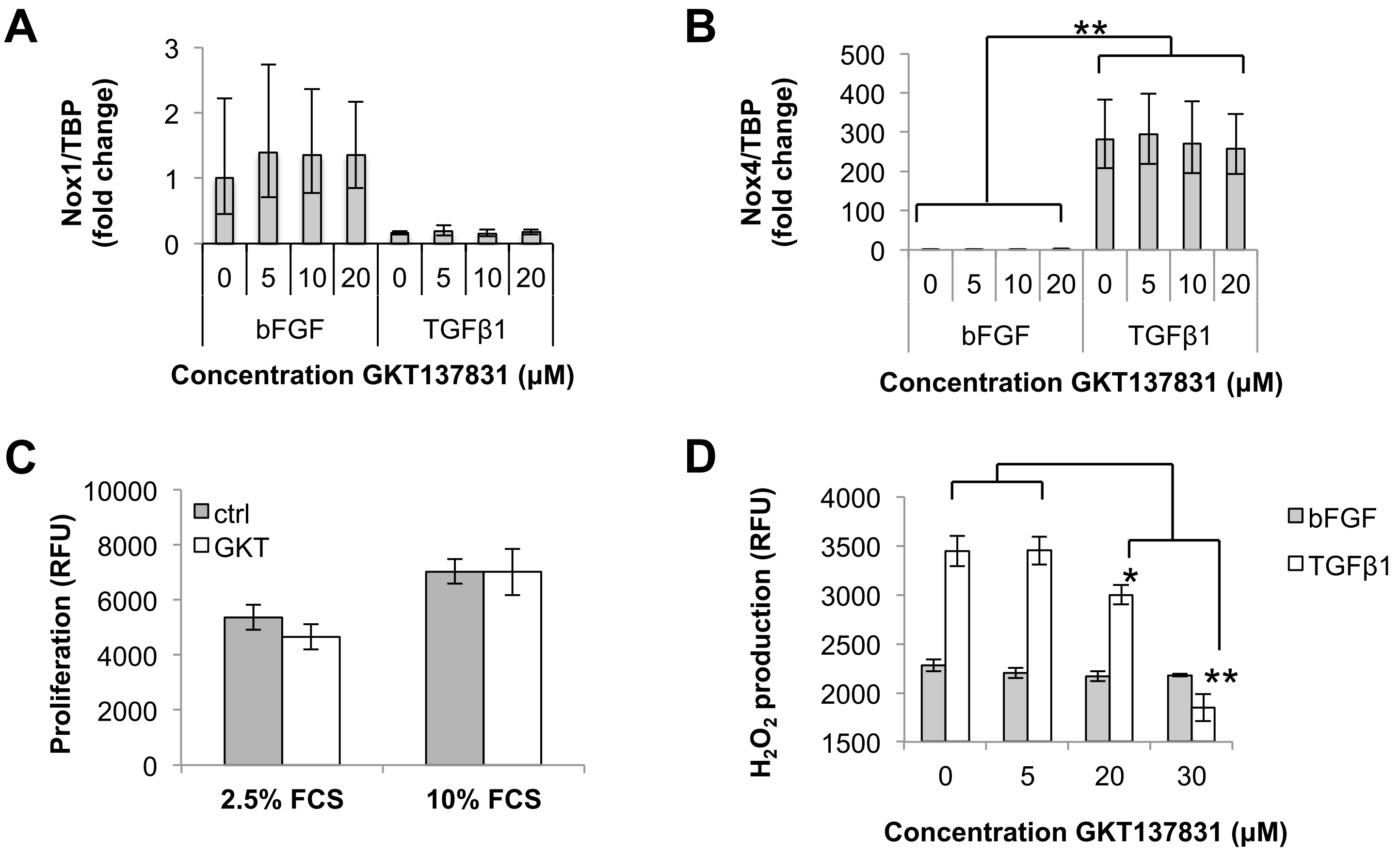

Supplemental Fig. S8 GKT137831 attenuates elevated H_2O_2 production associated with stromal activation in a dose-dependent manner

Primary prostate human fibroblasts were treated with bFGF or TGFβ1 in the presence of the indicated concentration of GKT137831. Equal vehicle (DMSO) concentration was maintained across all samples before determination of **(A)** Nox1 or **(B)** Nox4 mRNA levels 48 h later via qPCR relative to the housekeeping gene TBP or **(C)** extracellular H_2O_2 levels via Amplex Red assay 24 h later. **(D)** Proliferation of primary prostate human fibroblasts cultured in DMEM supplemented with the indicated concentration of fetal calf serum (FCS) was measured via SybrGreen staining 96 h after treatment with 30 µM GKT137831 or vehicle equivalent (ctrl, control). **(A-D)** Data represent mean ± SEM of at least four independent experiments using primary fibroblasts isolated from different donors. Statistical significance is shown (*, P <0.05; **, P <0.01).

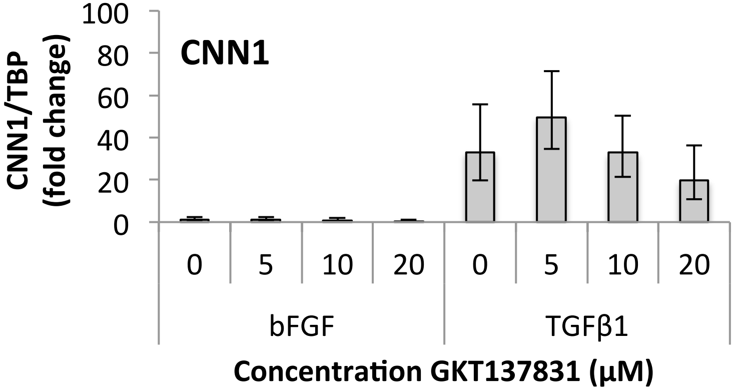

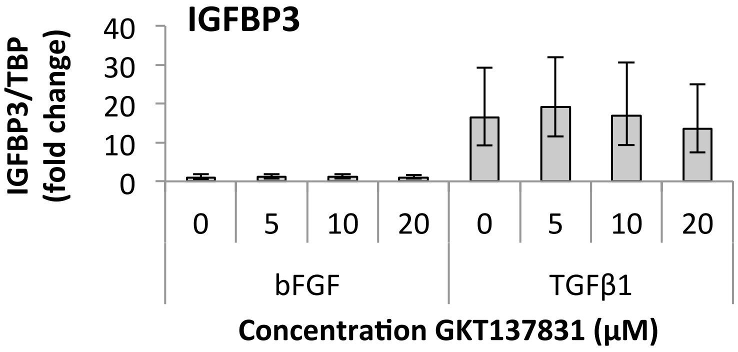

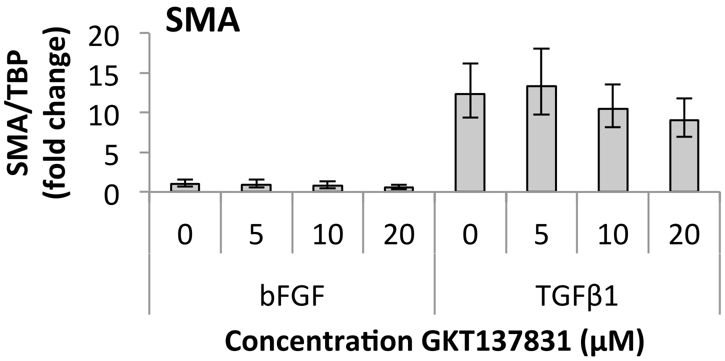

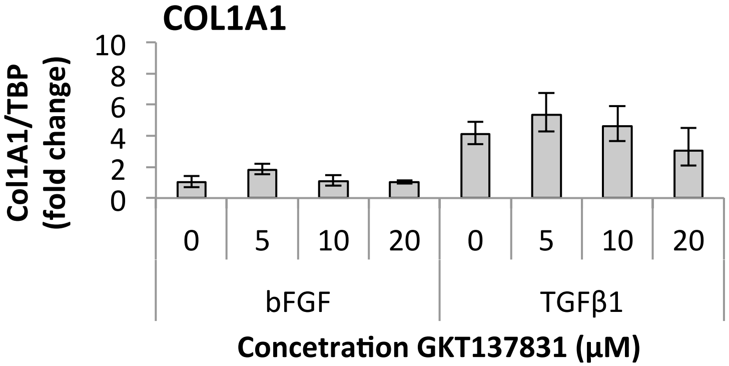

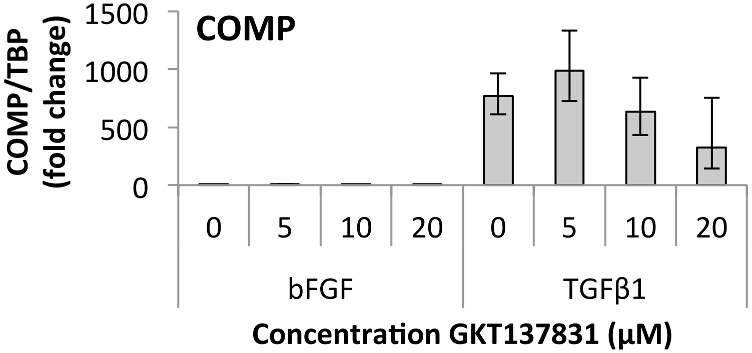

Supplemental Fig. S9 Dose dependent GKT137831 effects on fibroblast marker activation

Primary prostate human fibroblasts were treated with bFGF or TGFβ1 in the presence of the indicated concentration of GKT137831 for 48 h before qPCR of reactive stromal markers relative to the housekeeping gene TBP. Equal vehicle (DMSO) concentration was maintained across all samples. Data represent mean ± SEM of five independent experiments using primary fibroblasts isolated from different donors.

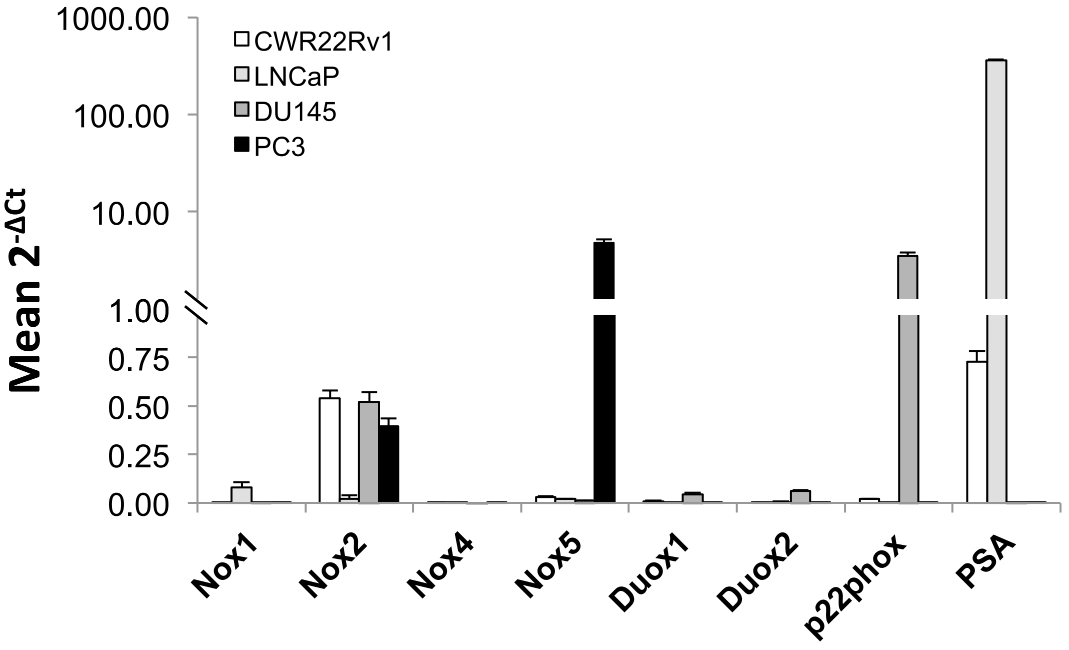

Supplemental Fig. S10 Expression levels of Nox enzymes in prostate cancer cell lines

qPCR of the 7 members of the Nox enzyme family and the accessory subunit p22phox relative to the housekeeping gene TBP in the indicated PCa cell lines cultured under routine conditions. PSA expression level for each cell line served as positive/negative control. Data represent mean + SEM of three independent experiments.

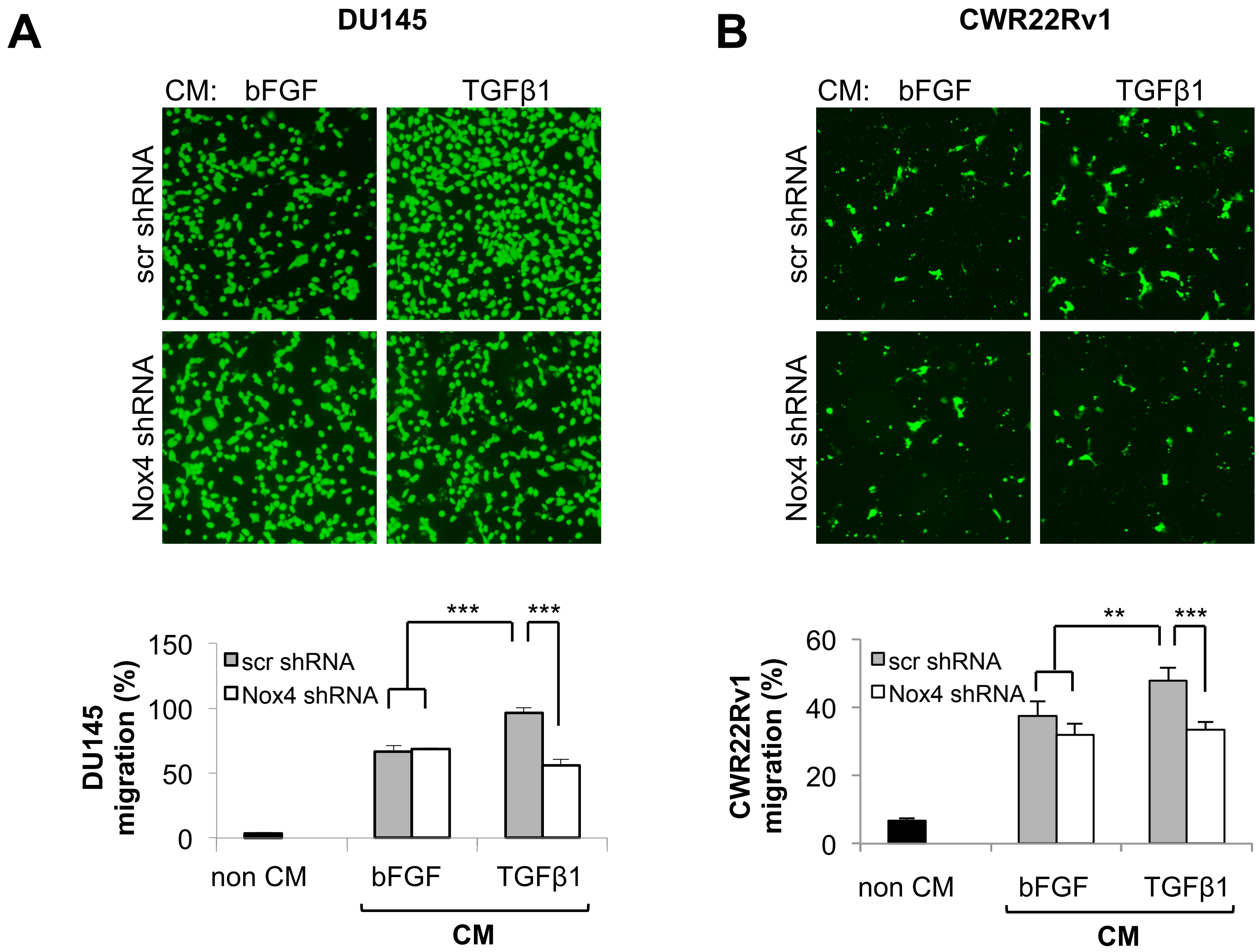

Supplemental Fig. S11 Nox4 knockdown attenuates increased migration of PCa cells induced by TGFβ1-mediated activated stromal fibroblasts

Nox4 was silenced via shRNA in primary human prostate fibroblasts before treatment with bFGF or TGFβ1 for 48 h. Cells were then switched to serum-free media for a further 48 h and the supernatant harvested as conditioned media (CM). CM or non-CM (serum-free DMEM incubated for the same duration without fibroblasts) was used as chemoattractant in the lower chamber of transwell migration assays for **(A)** DU145 or **(B)** CWR22Rv1 PCa cells seeded in serum-free media in transwell inserts and migrating cells analyzed **(A)** 24h or **(B)** 48h later. Data represent mean + SEM of three independent experiments using CM harvested from primary fibroblasts isolated from different donors. Representative images are depicted. Statistical significance is shown (**, $P < 0.01$; ***, $P < 0.001$).
