# Supplementary material for: Inhibition of Nox4‐dependent ROS signaling attenuates prostate fibroblast activation and abrogates stromal‐mediated protumorigenic interactions
Source: Int J Cancer. 2018 Mar 1;143(2):383–95. doi: 10.1002/ijc.31316 (PMC6067067; doi:10.1002/ijc.31316)
Supplement: Supplementary file 2 — Supporting Information Table 1 [file IJC-143-383-s002.pdf]

Supplemental Table 1 Taqman gene expression assays employed in this study

Gene	Taqman gene expression assay
CNN1	Hs00154543_m1
COL1A1	Hs00164004_m1
COMP	Hs00164359_m1
DUOX1	Hs00213694_m1
DUOX2	Hs00204187_m1
FAP	Hs00990806_m1
IGFBP3	Hs00426289_m1
NOX1	Hs00246589_m1
NOX2	Hs00166163_m1
NOX4	Hs00418356_m1
NOX5	Hs00225846_m1
p22phox	Hs03044361_m1
SMA	Hs01123712_m1

Gene	Custom designed Taqman primers and probes
PSA	Sense: 5'-GTCTGCGGCGGTGTTCTG-3'
	Antisense: 5'-TGCCGACCCAGCAAGATC-3'
	Probe: 5'-FAM-CACAGCTGCCCACTGCATCAGGA-TAMRA-3'
TBP	Sense: 5’-CACGAACCACGGCACTGATT-3’
	Antisense: 5’-TTTTCTGCTGCCAGTCTGGAC-3’
	Probe: 5’-FAM-TCTTCACTCTTGGCTCCTGTGCACA-TAMRA-3′
